# Structural, expression and evolutionary analysis of the non-specific phospholipase C gene family in *Gossypium hirsutum*

**DOI:** 10.1186/s12864-017-4370-6

**Published:** 2017-12-19

**Authors:** Jiuling Song, Yonghe Zhou, Juren Zhang, Kewei Zhang

**Affiliations:** 1 Ministry of Education Key Laboratory of Plant Cell Engineering and Germplasm Innovation, School of Life Science, Shandong University, Jinan, Shandong China; 2 School of Computer Science and Technology, Jilin University, Changchun, Jilin China

**Keywords:** ABA, Abiotic stress, Cloning, Evolution, Expression, *Gossypium hirsutum*, Nonspecific phospholipase C (NPC), Structure

## Abstract

**Background:**

Nonspecific phospholipase C (NPC), which belongs to a phospholipase C subtype, is a class of phospholipases that hydrolyzes the primary membrane phospholipids, such as phosphatidylcholine, to yield sn-1, 2-diacylglycerol and a phosphorylated head-group. NPC plays multiple physiological roles in lipid metabolism and signaling in plants. To fully understand the putative roles of *NPC* genes in upland cotton, we cloned *NPC* genes from *Gossypium hirsutum* and carried out structural, expression and evolutionary analysis.

**Results:**

Eleven *NPC* genes were cloned from *G. hirsutum*, which were found on chromosomes scaffold269.1, D03, A07, D07, A08, D11, and scaffold3511_A13. All GhNPCs had typical phosphoesterase domains and have hydrolase activity that acts on ester bonds. GhNPCs were annotated as phospholipase C, which was involved in glycerophospholipid metabolism, ether lipid metabolism, and biosynthesis of secondary metabolites. These *GhNPC*s showed differential expression patterns in distinct plant tissues and in response to various types of stress (low-phosphate, salt, drought, and abscisic acid). They also had different types and numbers of *cis*-element. GhNPCs could be classified into four subfamilies. Four pairs of *GhNPC*s were generated by whole-genome duplication and they underwent purifying selection.

**Conclusions:**

Our results suggested that *GhNPCs* are involved in regulating key abiotic stress responses and ABA signaling transduction, and they may have various functional roles for different members under complex abiotic stress conditions. Functional divergence may be the evolutionary driving force for the retention of four pairs of duplicate *NPCs*. Our analysis provides a solid foundation for the further functional characterization of the GhNPC gene family, and leads to potential applications in the genetic improvement of cotton cultivars.

**Electronic supplementary material:**

The online version of this article (10.1186/s12864-017-4370-6) contains supplementary material, which is available to authorized users.

## Background

Phospholipases, including patatin-related phopholipase A, phospholipase A_1_, phospholipase A_2_, phospholipase C, and phospholipase D, affect not only metabolism but also intracellular signaling [1, 2]. Phospholipase C, a subfamily of the phospholipase superfamily, can be divided into phosphatidylinositol-specific phospholipase C and nonspecific phospholipase C based on respective affinities to different substrates [3, 4]. The NPCs were discovered as a type of plant phospholipid-cleaving enzyme, one that preferentially hydrolyses phosphatidylcholine (PC) but which could also act on other lipids, such as phosphatidylethanolamine (PE) [1, 2, 4].

NPC was first discovered in the pathogenic bacterium *Clostridium perfringens* [5]. In 1955, an NPC-like activity was identified in plant plastid fractions [6]. Since then, NPC activity was discovered successively in peanut seeds [7], rice grains [8], tomatoes [9], cultured plant cells [10], and petunia flowers [11]. However, the analysis of NPC function in plants was hindered by insufficient molecular and genetic information. Eventually, in 2005, six *NPC* genes were identified from the Arabidopsis genome [12]. The subsequent analyses of NPCs in Arabidopsis indicated that NPCs play important and diverse roles in various processes. For example, NPC1 is involved in the plant response to heat [13] whereas NPC3 and NPC4 are important in BL-mediated signaling in root growth [14]. NPC4 is also involved in the plant response to phosphate starvation and plays a role in auxin signaling [12, 14]. NPC4 and its derived lipids positively modulate ABA response and promote plant tolerance to drought and salt stresses [15, 16]. NPC4 also plays a role in both early and long-term responses to Al stress [17]. NPC5 and diacylglycerol promote lateral root development under mild salt stress, and the former is involved in membrane glycerolipid remodeling during phosphate limitation in leaves of Arabidopsis [18, 19]. Taken together, these results clearly indicate that Arabidopsis NPCs are involved in many biological processes.

Although NPCs have been systematically investigated in Arabidopsis, only a few physiological functions and signaling roles have been clearly demonstrated at present. Moreover, there are few studies of NPCs in other plant species. Cotton, well known for its commercial significance both as a natural textile fiber and a vegetable oil yielding cultivar, offers a remarkable model system for learning more about polyploidization, cell elongation and cell wall biosynthesis [20, 21]. To fully understand the putative roles of *NPC* genes in plant, a systematic analysis for the NPC gene family in cotton is necessary.

In this study, we first cloned 11 *NPC* genes in *G. hirsutum*, and then carried out analyses of gene structure, conserved domains and motifs, secondary structure, three-dimensional structure, phylogeny, chromosomal location, and gene synteny. To investigate whether the expression of the *GhNPC* genes show tissue specificity, quantitative real-time PCR was used to study the expression patterns of these genes in various organs. To clarify their functions in response to various types of stress, the quantitative real-time PCR was also performed under four different treatments, namely low-phosphate, salt, drought, and abscisic acid (ABA). We also conducted *cis*-regulatory element identification and putative functional analysis. Finally, evolutionary analysis was carried out according to the above results. The results not only broaden our insight into the roles of *NPC* genes in plant abiotic stress responses and signaling transduction but also lay the foundation for further functional analyses of NPCs in cotton.

## Methods

### Identification of *GhNPC*s, *GaNPC*s, and *GrNPC*s

The *G. hirsutum* genome sequences were downloaded from the Cotton Research Institute of Nanjing Agricultural University (http://mascotton.njau.edu.cn, Gossypium_hirsutum_v1.1) and Institute of Cotton Resrarch of CAAS (http://cgp.genomics.org.cn/page/species/index.jsp#, Gossypium_hirsutum_v1.0), respectively. The *G. arboretum* genome (G.arboreum_BGI-A2_v1.0) and the *G. raimondii* genome (G.raimondii_JGI_221_v2.1) were downloaded from the CottonGen (https://www.cottongen.org/). Then, local databases were constructed. Arabidopsis NPC sequences obtained from The Arabidopsis Information Resource (TAIR, http://www.arabidopsis.org/) were used as queries against the local databases and the National Center of Biotechnology Information (NCBI). The choice of a candidate NPC was based on the E-value (1e^−5^). All the obtained sequences were put into SMART (http://smart.embl-heidelberg.de/) and InterPro (http://www.ebi.ac.uk/interpro/) to confirm the existence of the phosphoesterase domain [22]. The redundant sequences were then removed.

### Cloning and structural analysis of *GhNPC* genes

*GhNPCs* were amplified from the cDNA derived from the total RNA extracted from flowering stage cotton Zhong9807, cloned into the pGEM-T Easy Vector (TRANSGEN, Beijing, China), and sequenced in both directions to verify the gene sequences. The primers used for gene amplification are listed in Additional file 1: Table S1. The molecular weight (Mw) and isoelectric points (pI) of the GhNPCs were predicted by the online ExPASy proteomics server database (http://expasy.org/) [23]. The identification of exon/intron structures for each *GhNPC* gene was studied by comparing the sequences of CDS and genomic DNA. Diagrams of the exon-intron structures were produced by the Gene Structure Display Server 2.0 (GSDS; http://gsds.cbi.pku.edu.cn/) [24]. The conserved domains of NPC protein were examined by NCBI Conserved Domain search (https://www.ncbi.nlm.nih.gov/Structure/cdd/wrpsb.cgi) and SMART (http://smart.embl-heidelberg.de/) [25], and were located with HMMSCAN (https://www.ebi.ac.uk/Tools/hmmer/search/hmmscan) [26]. Conserved motifs of GhNPCs were analyzed by the MEME program (http://meme-suite.org/) using the following parameters: minimum motif width = 6; maximum motif width = 50; maximum motif number = 20; any number of repetitions; all of the other parameters were set at default values [27]. Furthermore, all identified motifs were annotated using the ScanProsite tool of ExPASy (http://prosite.expasy.org/scanprosite/) [28]. Secondary structures of GhNPCs were predicted using JPred4 server (http://www.compbio.dundee.ac.uk/jpred4) [29]. Three-dimensional structures were predicted using SWISS-MODEL (https://swissmodel.expasy.org/) [30]. Structure of *Francisella tularensis* Acid Phosphatase A (AcpA) bound to orthovanadate (2d1g.1.A) was used to build a structural model for the GhNPCs [1, 31].

### Multiple sequence alignments and phylogenetic tree construction

Multiple sequence alignments of GhNPCs were performed using the software MEGA7 and tinted using DNAman [32]. To gain insights into the phylogenetic relationships between NPC in cotton and other plant species, the phylogenetic tree was constructed with the software MEGA7 that used the Maximum-likelihood (ML) method based on the WAG amino acid substitution model [32]. A bootstrap analysis was employed with 1000 replicates. The protein sequences used to generate a phylogenetic tree were from *G. hirsutum* (11), *G. arboreum* (7), *G. raimondii* (11), *Arabidopsis thaliana* (6)*, Glycine max* (7), *Oryza sativa* (5), *Sorghum bicolor* (5), *Physcomitrella patens* (5), and *Selaginella moellendorffii* (2) (sequences are given in Additional file 2: Data Set S1).

### Syntenic analysis

MCscan X was employed to identify homologous genes among *G. hirsutum*, *G. raimondii* and *G. arboreum*, respectively [33]. The chromosomal distribution of *NPC*s and syntenic blocks were drawn by Circos-0.69 (http://circos.ca/) [34]. To evaluate the selective pressure over duplicate genes, non-synonymous (*K*_*a*_) and synonymous (*Ks*) substitution were calculated using MCscan X [33].

### *Cis*-regulatory element identification

The promoters of the *GhNPC*s, which were 1.5 kb upstream from the transcriptional start site, were mapped onto the *G. hirsutum* genome. The *cis*-acting regulatory elements were identified using Plant CARE (http://bioinformatics.psb.ugent.be/webtools/plantcare/html/) [35]. Among *cis*-acting regulatory elements that we identified, we chose abiotic stress response and phytohormone-related elements for the expression analysis.

### Plant material and treatments for expression analysis

Upland cotton (*G. hirsutum*, Zhong9807) seeds were grown in a greenhouse at 30 °C day/25 °C night and 60−70% relative humidity, under a 14-h light/10-h dark photoperiod with a photon flux density (PFD) of 800 μmol m^−2^ s^−1^. Zhong9807 is a typically salt-tolerant variety [36]. For the expression analysis of *GhNPC*s in different plant tissues, we used cotton grown in soil as material. Samples of taproot, lateral root, stem, cotyledon, senescent leaf, expanded leaf, and shoot tip were collected at the six-leaf stage; bract, petal, and ovule tissues were harvested on the first day of flowering. For the expression analysis of *GhNPCs* under various abiotic or exogenous hormone stresses, we used cotton grown hydroponically as material. Control plants were grown in Hoagland’s solution. We transferred cotton seedlings at the six-leaf stage randomly into various stress solutions: low-phosphate (5 μM), salt (200 mM NaCl), or drought (20% PEG6000). For the hormone treatment, 200 μM of ABA was used [37]. The roots were collected at 0, 1, 2, 3, 6, 9 and 12 h. All tissues were immediately frozen in liquid nitrogen and stored at −80°C for later use.

### Quantitative real-time PCR

Total RNA was extracted from the different plant tissues with EASYspin TRIzol reagent (TIANGEN, Beijing, China). The cDNA was synthesized using the PrimeScript RT Reagent Kit (TAKARA, Dalian, China), and 600 ng total RNA were used. The cDNA samples were diluted 10-fold to serve as the templates for the subsequent PCR analyses.

Real-time quantitative PCR analyses were carried out in the LightCycler® 96 System (Roche Diagnostics Corporation, Indianapolis, USA) with the SYBR Premix Ex Taq II (TAKARA, Dalian, China) in a 10-μl reaction volume, which contained 5 μl of SYBR Premix Ex Taq II, 0.4 μl of each forward and reverse primer (Additional file 3: Table S2), 0.8 μl of diluted cDNA template, and an appropriate amount of sterile double-distilled water. The conditions for amplification were as follows: 30 s at 95 °C, 40 cycles of 5 s at 95 °C, and 30 s at 60 °C. With the *Histone* gene (GenBank accession number NC_006639) used as the reference gene [38, 39], the expression level was calculated using the 2^−ΔΔCt^ (ΔΔ*C*_T_ = (*C*_T, *GhNPC*s_ - *C*_T, *Histone*_) _treatments at different times_ - (*C*_T, *GhNPC*s_ - *C*_T, *Histone*_) _0 h_) method for abiotic stress treatments and the 2^-ΔCt^ (Δ*C*_T_ = *C*_T, *GhNPC*s_ - *C*_T, *Histone*_) method for various tissues [40]. The entire experiment was repeated three times.

## Results

### Identification of *GhNPC*s, *GaNPC*s, and *GrNPC*s

After merging results from the above strategies, eleven *NPC* genes were identified from Gossypium_hirsutum_v1.1 genome and nine *NPC* genes were identified from Gossypium_hirsutum_v1.0 genome, respectively. While, seven *NPC*s were identified from *G. arboretum* genome and were designated as *GaNPC1a*, *GaNPC1b*, *GaNPC2*, *GaNPC3*, *GaNPC4*, *GaNPC6a*, and *GaNPC6b* based on Arabidopsis *NPC* sequences, and eleven *GrNPC*s were identified from *G. raimondii* genome and were designated as *GrNPC1*, *GrNPC2*, *GrNPC3a*, *GrNPC3b*, *GrNPC3c*, *GrNPC4a*, *GrNPC4b*, *GrNPC6a*, *GrNPC6b*, *GrNPC6c*, and *GrNPC6d* based on Arabidopsis *NPC* sequences.

### Cloning and annotation of *NPC* genes family in cotton

**Fig. 1:**
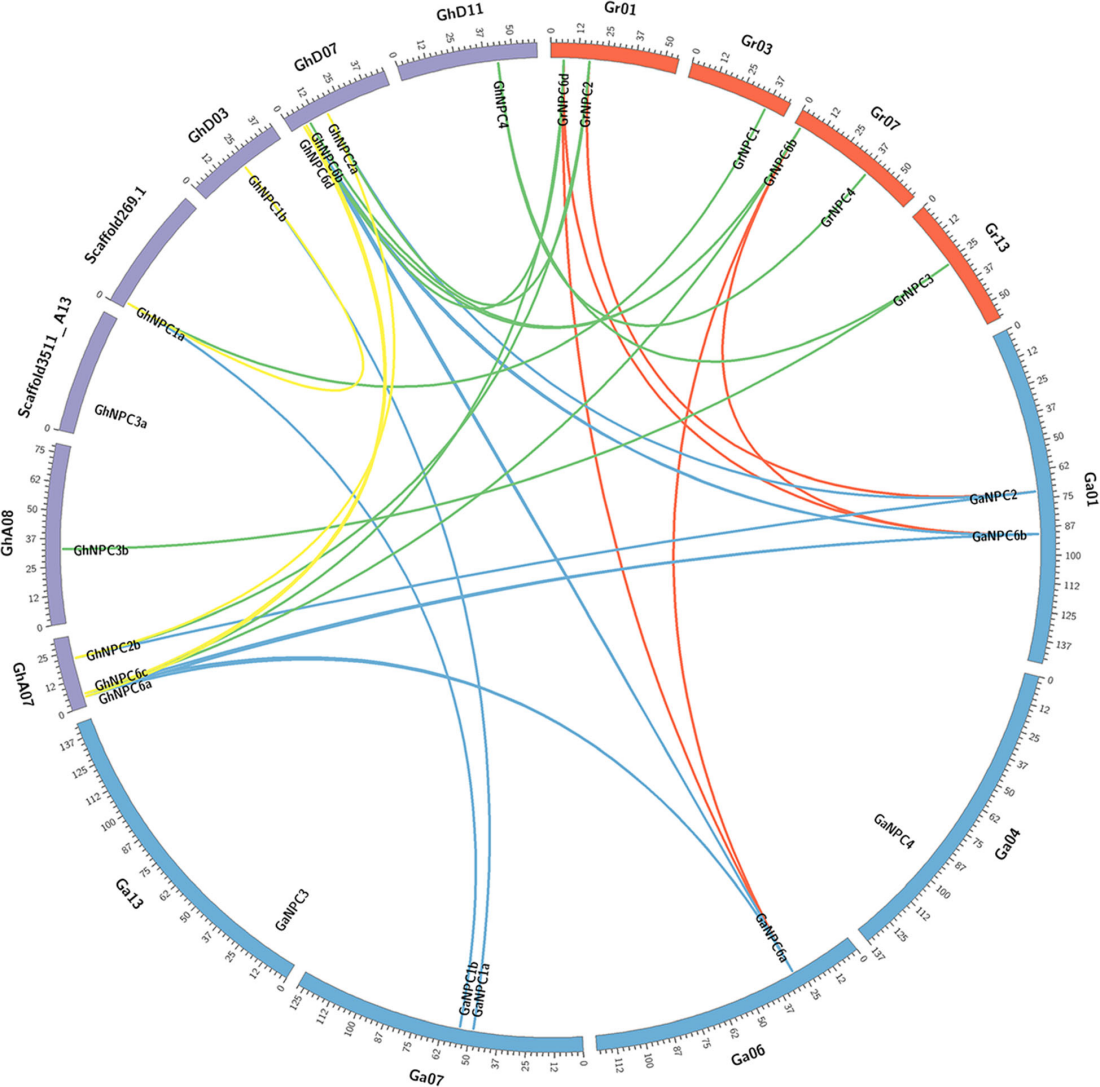
Syntenic block among *NPC*s from *G. hirsutum*, *G. arboretum* and *G. raimondii*. The chromosomes of *G. hirsutum*, *G. arboretum* and *G. raimondii* are indicated in purple, blue and red, respectively. Yellow lines indicate segmental duplicated pairs of *GhNPC*s. The putative orthologous *NPC*s between *G.hirsutum* and *G. arboretum*, *G. hirsutum* and *G. raimondii*, *G. arboretum* and *G. raimondii* are connected by blue, green and red lines, respectively

Finally, a total of 11 *GhNPC* genes were cloned and were designated as *GhNPC1a*, *GhNPC1b*, *GhNPC2a*, *GhNPC2b*, *GhNPC3a*, *GhNPC3b*, *GhNPC4*, *GhNPC6a*, *GhNPC6b*, *GhNPC6c*, and *GhNPC6d*, on the basis of sequence similarities to Arabidopsis *NPC*s. Among them, sequences of five *GhNPC*s (*GhNPC2b*, *GhNPC3a*, *GhNPC4*, *GhNPC6c*, and *GhNPC6d*) are consistent with the Gossypium_hirsutum_v1.1 genomic data; sequences of three *GhNPC*s (*GhNPC1b*, *GhNPC3b*, *GhNPC6a*) are consistent with the Gossypium_hirsutum_v1.0 genomic data; *GhNPC1a* has 1 base difference with Gossypium_hirsutum_v1.0 genomic data, but the amino acid encoded has not changed; *GhNPC2a* has 4 base differences with Gossypium_hirsutum_v1.1 genomic data, but the amino acid encoded has not changed; *GhNPC6b* has 3 base differences with Gossypium_hirsutum_v1.0 genomic data, and two amino acid changes (sequences are given in Additional file 4: Data Set S2). As shown in Table 1, these GhNPC proteins varied considerably in their length, Mw, and pI. Protein length ranged from 305 to 560 amino acids (aa), with the majority (81.82%) of proteins containing 488–520 aa. Mw ranged from 34.64 to 63.80 kDa and the pI ranged from 5.18 to 6.84 pH; however, all members had pI values <7 (Table 1). Based on the genomic data, we located 11 *GhNPCs* on chromosomes. The *GhNPC* genes were unevenly distributed over the *G. hirsutum* genome, with chromosomes A07 and D07 each having three genes and five other chromosomes (scaffold269.1, D03, A08, D11 and scaffold3511_A13) having one gene each (Table 1 and Fig. 1).

**Table 1:** The characteristics of *NPC* genes from *G. hirsutum*

Gene name	Locus	Strand	ORF length (bp)	Protein			Intron number
				Length (aa)	Mw (kDa)	pI	
*GhNPC1a*	scaffold269.1:65,602-67,720	–	1461	486	54.52	6.37	2
*GhNPC1b*	D03: 22,587,094-22,589,215	–	1461	486	54.51	6.37	2
*GhNPC2a*	D07:17,824,914-17,829,009	+	1563	520	58.71	6.44	3
*GhNPC2b*	A07:22,144,436-22,148,517	+	1548	515	58.13	6.45	3
*GhNPC3a*	scaffold3511_A13:74,984-69,115	–	1527	508	56.96	5.18	3
*GhNPC3b*	A08: 33,048,822-33,049,930	–	918	305	34.64	5.76	2
*GhNPC4*	D11:43,575,117-43,570,466	–	1683	560	63.80	6.38	2
*GhNPC6a*	A07: 4,310,983-4,312,741	+	1569	522	58.04	6.68	2
*GhNPC6b*	D07: 6,984,026-6,985,784	+	1569	522	58.17	6.84	2
*GhNPC6c*	A07:5,762,622-5,765,615	+	1530	509	56.64	6.74	2
*GhNPC6d*	D07:5,782,790-5,785,614	+	1530	509	56.80	6.74	2

*Mw* molecular weight, *pI* isoelectric points

### Structure analysis of GhNPCs

**Fig. 2:**
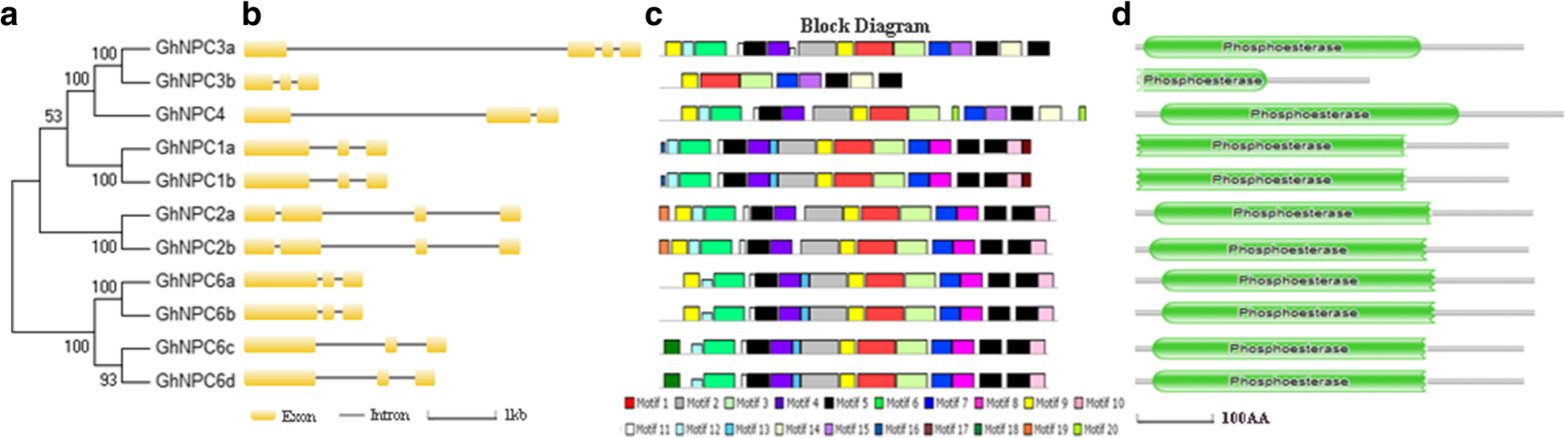
Exon-intron structure, conserved motif and conserved domain analysis of *GhNPC* genes. **a**. Phylogenetic tree of GhNPCs. Phylogenetic tree was generated using MEGA7 by the Maximum Likelihood method based on the protein sequences of GhNPCs. **b**. Exon-intron structures. Exons and introns of *G. hirsutum* are shown as yellow rounded rectangles and thin lines, respectively. **c**. Conserved motif analysis. Twenty motifs were identified. Different color boxes represent different types of motifs. Sequence information of twenty motifs was shown in Additional file 8: Table S5. **d**. Conserved domain analysis. The conserved domains of NPC protein were examined by NCBI Conserved Domain search and SMART, and were located with HMMSCAN

**Fig. 3:**
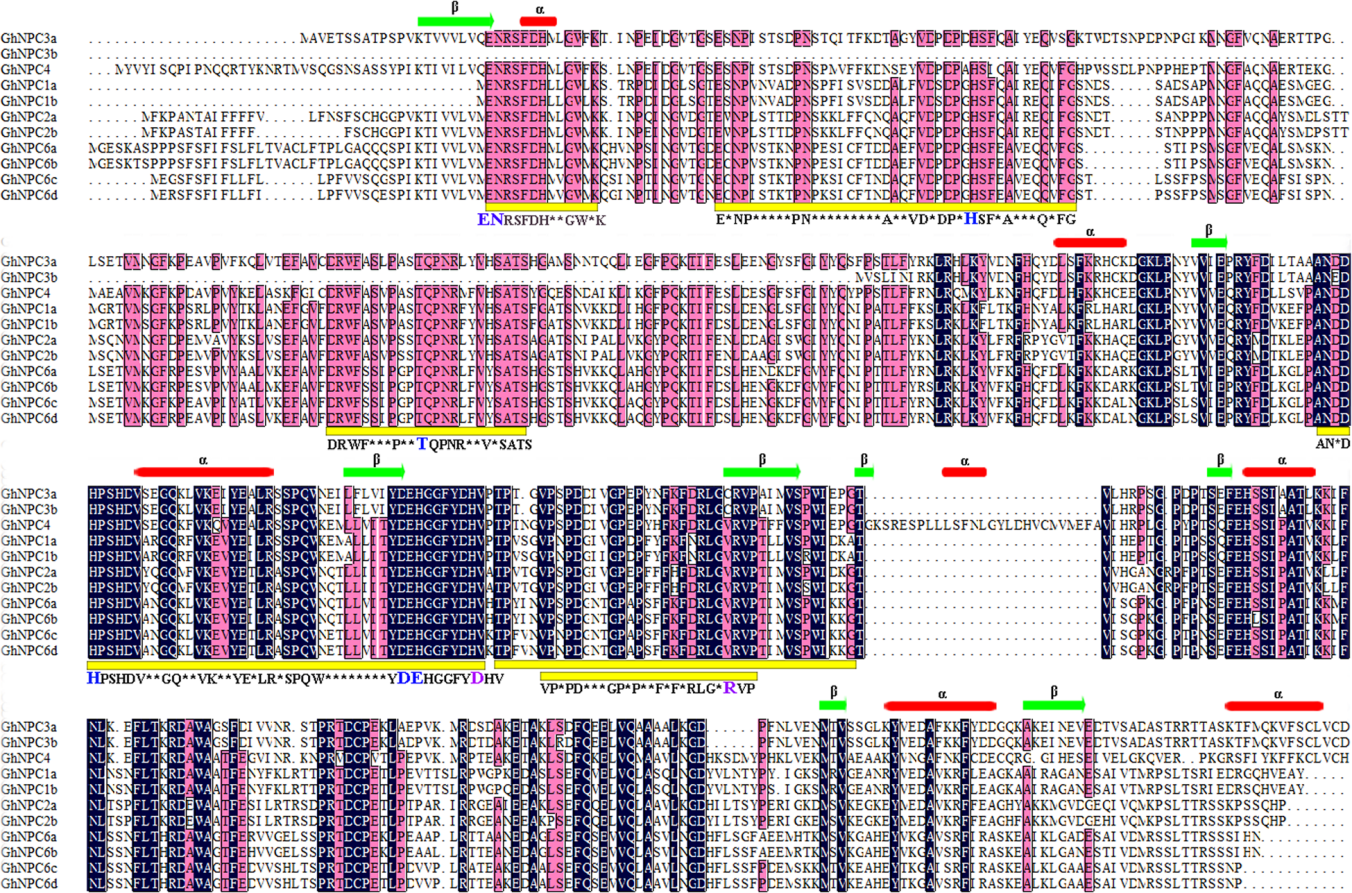
Alignments of GhNPCs. Multiple sequence alignments of GhNPCs were performed using the software MEGA7 and tinted using DNAman. Six motifs which were annotated as the phosphoesterase domain were marked in yellow. Putative active site (marked in blue) and conserved Asp-Arg ion pair (marked in purple) was indicated according to Pokotylo et al. [1]. Secondary structures of GhNPCs were predicted using JPred4 server [29]. Beta sheet were marked in green arrow and alpha helices were marked in red rectangle

**Fig. 4:**
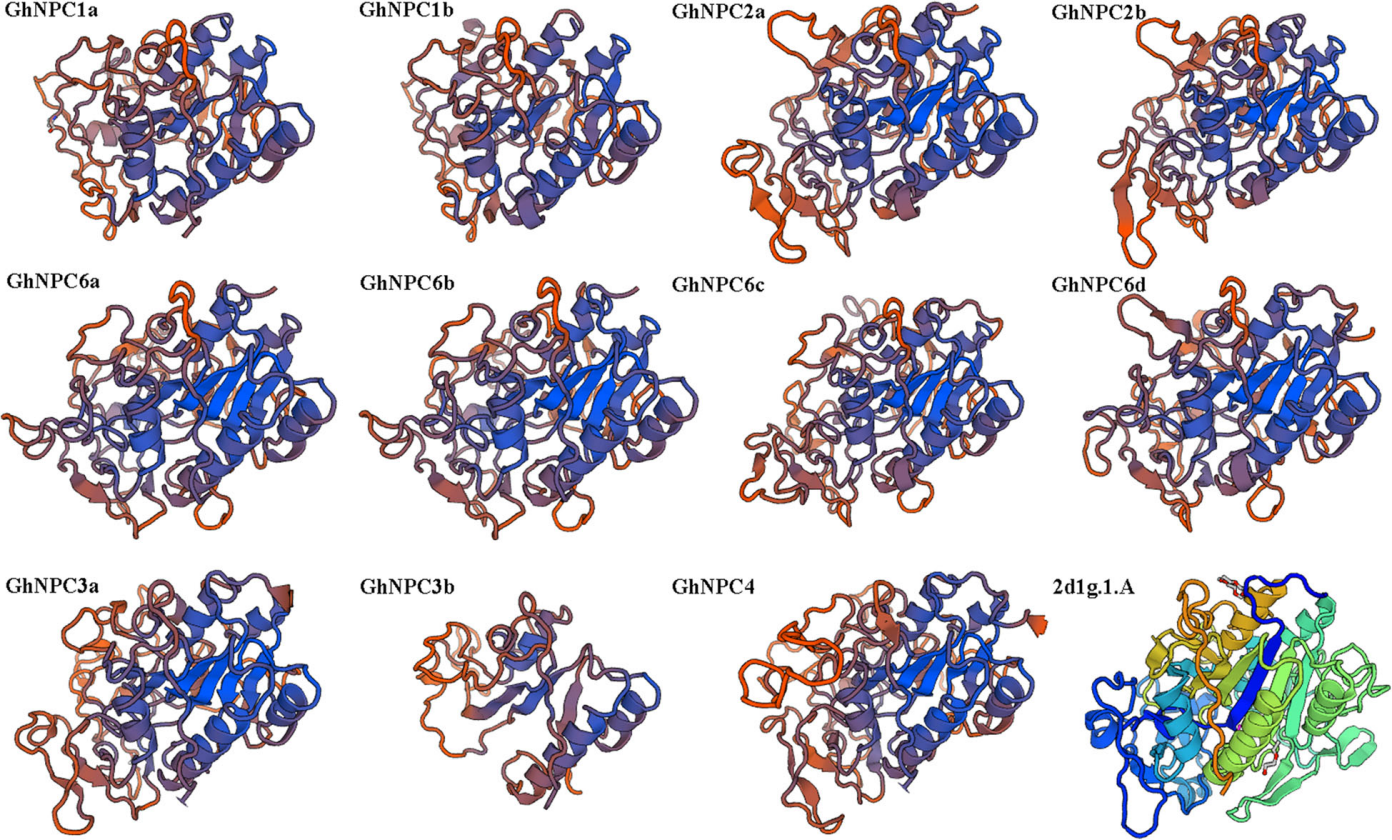
Three-dimensional structure of GhNPCs. Three-dimensional structures were predicted using SWISS-MODEL [30]. Structure of *Francisella tularensis* Acid Phosphatase A (AcpA) bound to orthovanadate (2d1g.1.A) are used to build a structural model for the GhNPCs [1, 31]

The identification of exon/intron structures for each *GhNPC* gene was studied by comparing the sequences of CDS and genomic DNA [24]. Most of them had two introns, though three of them (*GhNPC2a*, *GhNPC2b*, and *GhNPC3a*) had three introns (Fig. 2b). The *GhNPC*s had preserved a relatively simple constant exon-intron composition. The conserved domains of the NPC protein were examined by the NCBI Conserved Domain search, SMART and HMMSCAN [25, 26]. All of these GhNPCs contained the phosphoesterase domain (Fig. 2d), which has hydrolase activity that acts on the ester bonds. Conserved motifs of GhNPCs were analyzed using the MEME program [27]. Among the GhNPCs the number of motifs ranged from 8 to 17 (Fig. 2c). As shown in Fig. 2c, most of the GhNPC members in the same subgroup shared common motif compositions. Six motifs — motif 1, motif 2, motif 3, motif 4, motif 6, and motif 9 — were annotated as the phosphoesterase domain (Fig. 3). As shown in Fig. 2c, present in all sequences were the motifs 1, 3, 5, 7, and 9. The majority of the GhNPCs contained the motif 8 and the motif 10, with the exception of NPC3 and NPC4. However, both motif 14 and motif 15 only existed in NPC3 and NPC4. Additionally, the motif 16 and the motif 17 were unique to subfamily NPC1; motif 19 was unique to subfamily NPC2; motif 20 was unique to NPC4; motif 18 was unique to NPC6c and NPC6d. Some motifs, such as the motif 13, were shared in NPC1 and NPC6. Additional, we also predicted secondary structures using JPred4 [29], and three-dimensional structures using SWISS-MODEL [30]. As shown in Fig. 3 and Fig. 4, the structures of GhNPCs were composed by the beta sheet and several alpha helices.

### Phylogenetic and syntenic analysis

**Fig. 5:**
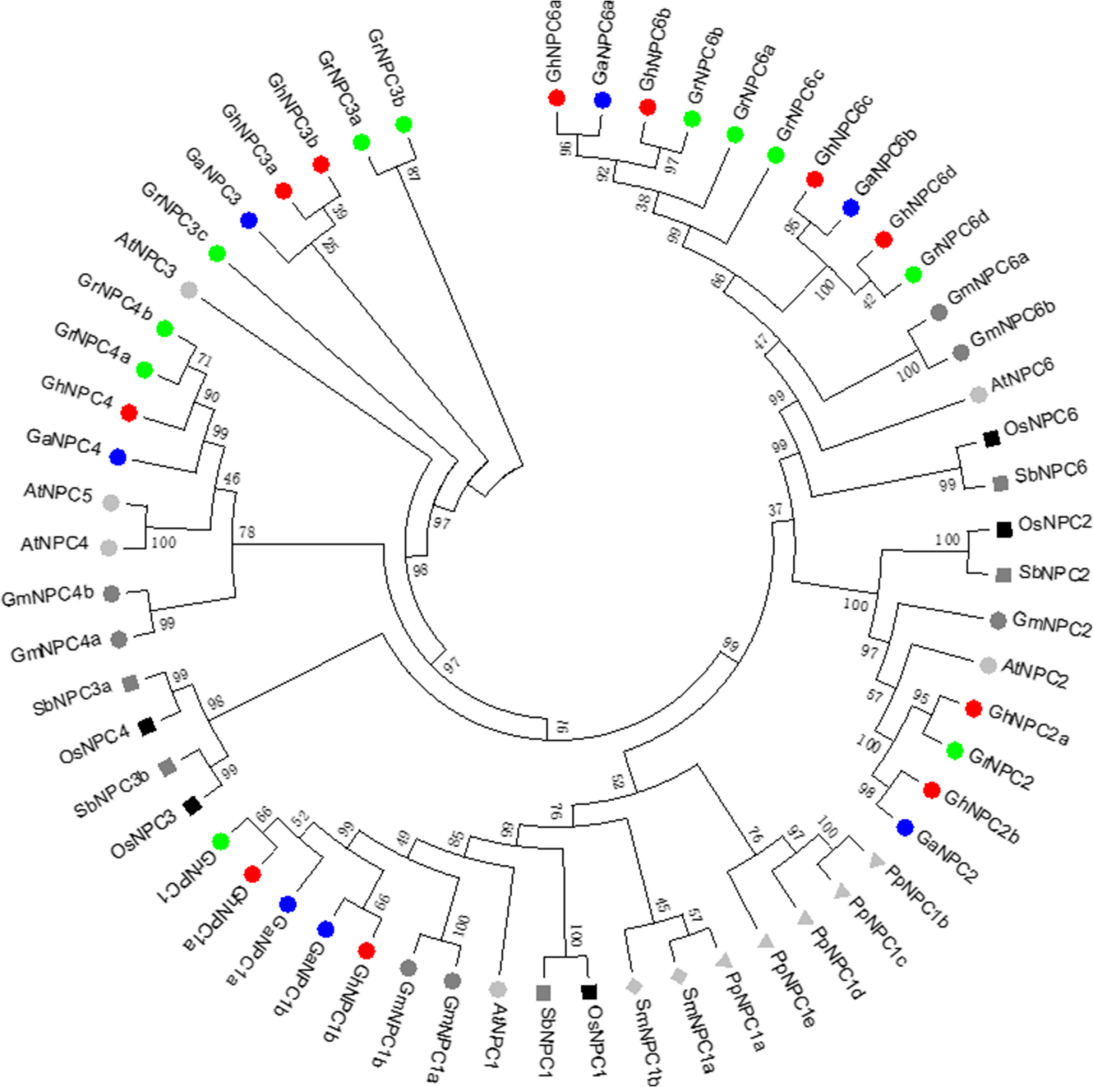
Phylogenetic tree of plant NPC. The tree was constructed using MEGA7 using the Maximum Likelihood method. Branches corresponding to partitions reproduced in less than 50% bootstrap replicates are collapsed. The bootstrap consensus tree inferred from 1000 replicates is taken to represent the evolutionary history of the taxa analyzed. Initial tree(s) for the heuristic search were obtained automatically by applying Neighbor-Join and BioNJ algorithms to a matrix of pairwise distances estimated using a JTT model, and then selecting the topology with superior log likelihood value. The analysis involved 59 amino acid sequences. Species abbreviations: At, *Arabidopsis thaliana*; Gm, *Glycine max*; Os, *Oryza sativa*; Pp, *Physcomitrella patens*; Sb, *Sorghum bicolor*; Sm, *Selaginella moellendorffii*; Gh, *Gossypium hirsutum*; Ga, *G. arboretum*; Gr, *G. raimondii*; NPC, non-specific phospholipase C

To gain insight into the evolution of plant NPCs, we performed a phylogenetic analysis of the NPC protein sequences known from several evolutionarily distinct plant species. In addition to its putative ancestral species, *G. raimondii* and *G. arboreum*, we also analyzed two other dicot species, Arabidopsis and soya (*G. max*); two monocotyledon species, rice (*O. sativa*) and sorghum (*S. bicolor*); a moss (*P. patens*) and a lycophyte (*S. moellendorffii*) (Additional file 2: Data Set S1). The phylogenetic analysis suggested that NPCs could be grouped into four clusters: I (NPC1), II (NPC2), III (NPC3, NPC4, and NPC5) and IV (NPC6) (Fig. 5). However, except for Arabidopsis, NPC5 was neither identified in the dicot soya (*G. max*) and cotton (*G. hirsutum*, *G*. *raimondii* and *G. arboreum*) nor in any of the other four species (*O. sativa*, *S. bicolor*, *P. patens*, and *S*. *Moellendorffii*). Syntenic analysis showed that four pairs of *GhNPC* (*GhNPC1a* and *GhNPC1b*, *GhNPC2a* and *GhNPC2b*, *GhNPC6a* and *GhNPC6b*, *GhNPC6c* and *GhNPC6d*) were segmental duplicates. *GrNPCs* and *GaNPCs* (except *GaNPC3* and *GaNPC4*) had orthologous gene in *G. hirsutum* (Fig. 1). According to the results of phylogenetic and syntenic analysis, we found that *GhNPC1a*, *GhNPC2a*, *GhNPC4*, *GhNPC6b* and *GhNPC6d* were native to *G. raimondii*, while *GhNPC1b*, *GhNPC2b*, *GhNPC6a* and *GhNPC6c* were native to *G. arboretum*. In addition, the *K*a/*K*s ratio of four *G. hirsutum* duplicated gene pairs (*GhNPC1a* and *GhNPC1b*, *GhNPC2a* and *GhNPC2b*, *GhNPC6a* and *GhNPC6b*, *GhNPC6c* and *GhNPC6d*) were less than 1 (Additional file 5: Table S3), which suggested that they experienced purifying selection.

### Analysis of *cis*-elements in the *GhNPC* gene promoters

**Fig. 6:**
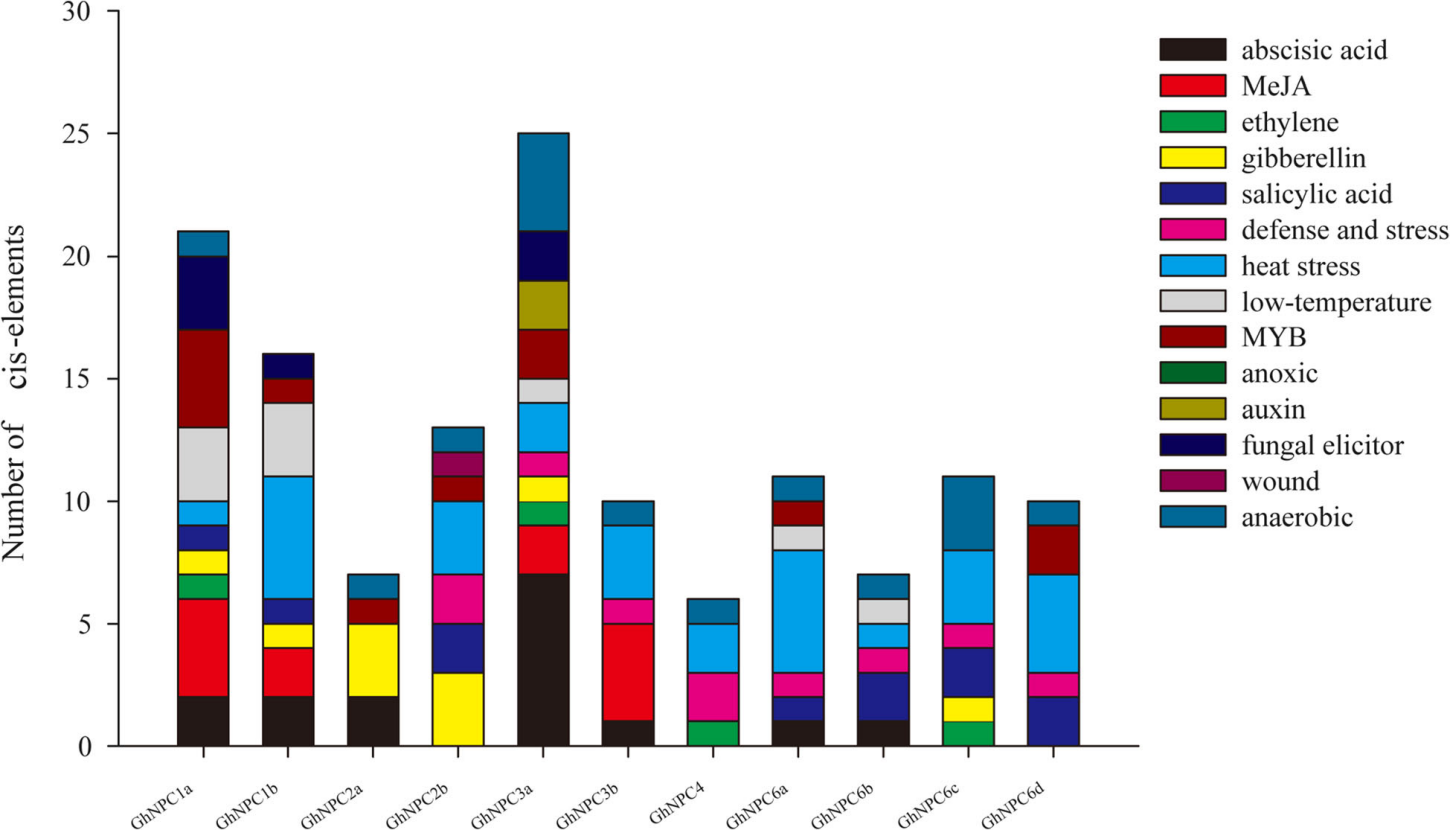
*Cis*-elements in the promoters of *GhNPC* genes that are related to stress responses. Different *cis*-elements with the same or similar functions are represented with the same color

To further explore gene function and regulation patterns, the *cis*-elements in *GhNPC* gene promoter sequences were studied. Regions of 1500 bp upstream from the start codons of each *GhNPC* gene were determined using the Plant CARE [35]. The results showed that the *cis*-elements could be divided into four major classes: stress-responsive, hormone-responsive, development-related, and light-responsive (Additional file 6: Excel S1). Nine stress-responsive *cis*-elements were identified—HSE, MBS, MBSII, CCAAT-box, LTR, TC-rich repeats, ARE, WUN-motif, and Box-W1—which reflected plant responses to heat, drought, low-temperature defense stresses, anaerobic induction, wound-responsive, and fungal elicitors (Fig. 6 and Additional file 6: Excel S1). Ten kinds of hormone-responsive *cis*-elements were identified, such as abscisic acid-ABA, salicylic acid-SA, methyl jasmonate-MeJA, gibberellin-GA, auxin-IAA, and ethylene (Fig. 6 and Additional file 6: Excel S1). A relatively large number of light-responsive *cis*-elements in *GhNPC* promoters were found (Additional file 6: Excel S1). As shown in Fig. 6, different GhNPCs had different types and numbers of *cis*-element.

### Expression profiles of *GhNPC* genes in different plant tissues

**Fig. 7:**
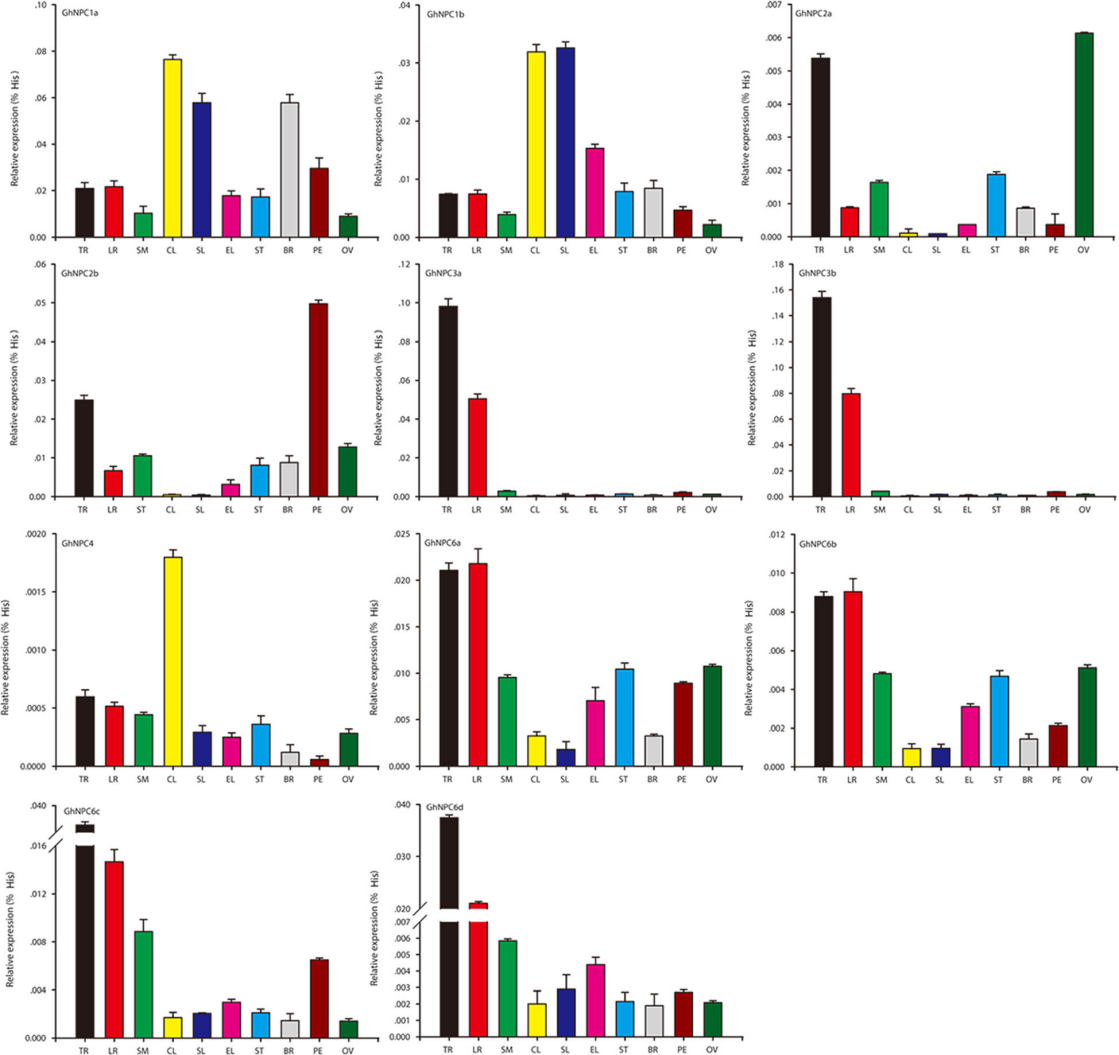
Expression of *GhNPC*s in various organs. The expression levels of *GhNPC*s were compared with *Histone,* wherein the expression level of *Histone* was defined as “1.” Note that the expression levels of different *GhNPC*s are scaled differently. Abbreviations: TR, taproot; LR, lateral root; SM, stem; CL, cotyledon, SL, senescent leave; EL, expanded leave; ST, shoot tip; BR, bract; PE, petal; OV, ovule. The expression level was calculated using the 2^-ΔCt^ (Δ*C*_T_ = *C*_T, *GhNPC*s_ - *C*_T, *Histone*_) method [38]. The entire experiment was repeated three times

To investigate the expression patterns of *GhNPC* genes in different organs and to determine their function in organ development, expression profiles were analyzed for 10 different tissues (taproot, lateral root, stem, cotyledon, senescent leaf, expanded leaf, shoot tip, bract, petal, and ovule) of the cotton cultivar ‘Zhong9807’ using real-time quantitative PCR. The mean threshold cycle (*C*_T_) of the reference gene (*Histone*) was 18.32. As shown in Fig. 7, *GhNPC1* and *GhNPC6* were ubiquitously expressed, whereas the others displayed tissue-specific expression patterns. *GhNPC2a* was mainly expressed in taproot and ovule. *GhNPC2b* was highly expressed in taproot and petal. *GhNPC3a* and *GhNPC3b* were mainly expressed in root. In addition, *GhNPC4* were expressed weakly in all of the 10 tissues, though it was relatively higher in cotyledon.

### Expression patterns of *GhNPC* genes in response to abiotic treatments

**Fig. 8:**
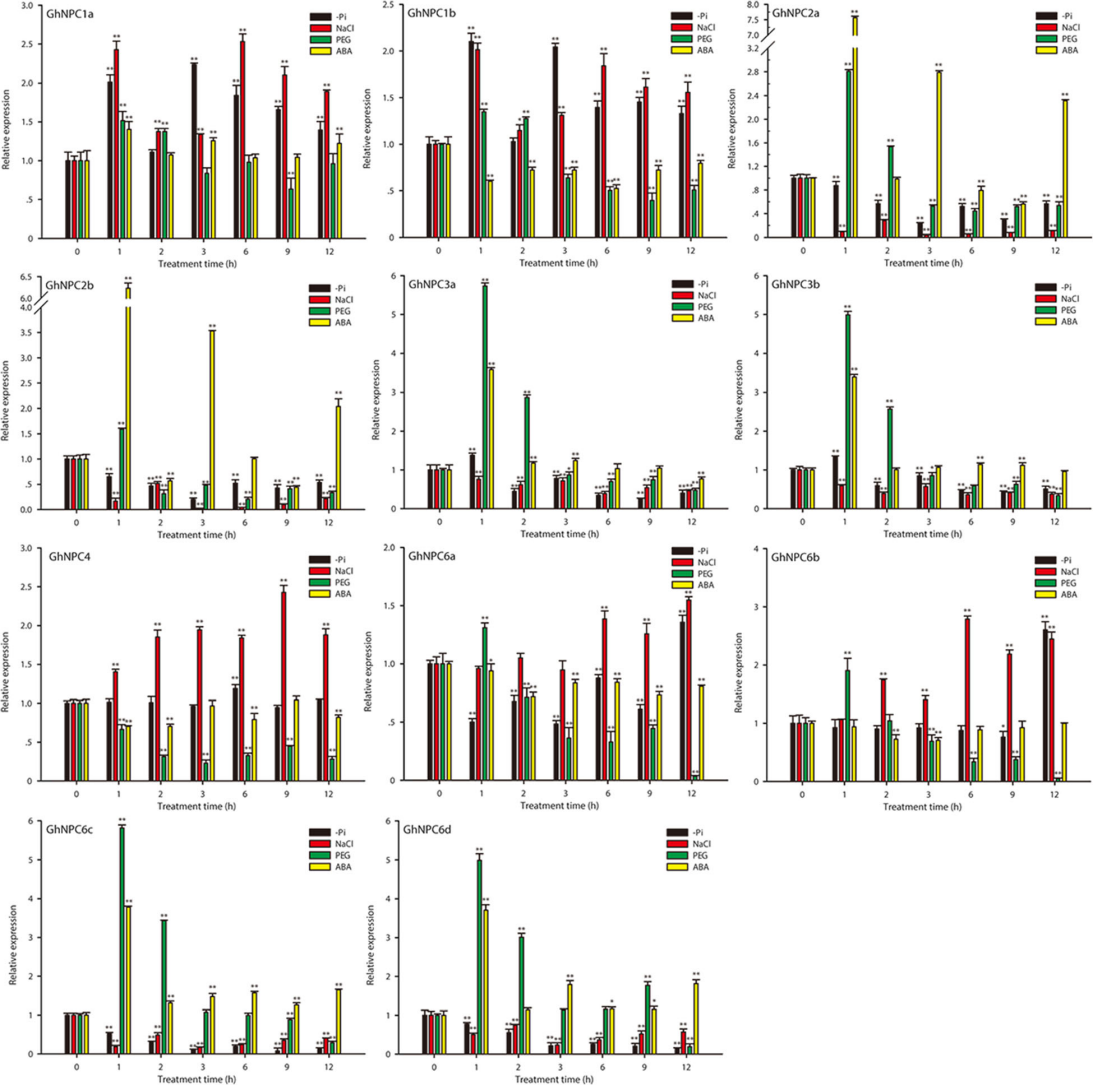
Expression of *GhNPC*s in root under various stresses. The expression levels of *GhNPC*s, which are shown in Y-axis, were compared with the control (0 h). Note that the expression levels of different *GhNPC*s are scaled differently. The expression level was calculated using the 2^−ΔΔCt^ (ΔΔ*C*_T_ = (*C*_T, *GhNPC*s_ - *C*_T, *Histone*_) _treatments at different times_ - (*C*_T, *GhNPC*s_ - *C*_T, *Histone*_) _0 h_) method [38]. The entire experiment was repeated three times

To gain further insight into the role of the *GhNPC* genes under abiotic stress, we analyzed their expression profiles in response to low-phosphate, salt, drought, and ABA. As shown in Fig. 8, expression of the *GhNPC* genes was significantly affected by these stress treatments.

*GhNPC1a* and *GhNPC1b* were up-regulated under the treatments of low-phosphate and salt, up-regulated at 1 h and 2 h but then down-regulated under the drought treatment. Under the ABA treatment, *GhNPC1a* was slightly up-regulated but *GhNPC1b* was down-regulated.

Both *GhNPC2a* and *GhNPC2b* were obviously induced at 1, 3, and 12 h under the ABA treatment, whereas they were down-regulated under the treatments of low-phosphate and salt. Under the drought treatment, both genes up-regulated at first and then down-regulated.

*GhNPC3a* and *GhNPC3b* were both down-regulated under the salt treatment. Under the treatments of low-phosphate and drought, both genes were up-regulated at first and then down-regulated. Under the ABA treatment, *GhNPC3a* was up-regulated at first and down-regulated at 12 h, and *GhNPC3b* was also up-regulated at first and had no significant differential change at 12 h. Expression of *GhNPC4* was up-regulated under the salt treatment, but showed down-regulated under the drought treatment.

Expression of *GhNPC6a* and *GhNPC6b* were up-regulated at most time points under the salt treatment, yet down-regulated under the ABA treatment. By contrast, *GhNPC6a* and *GhNPC6b* were down-regulated at the beginning but up-regulated at 12 h under the low-phosphate treatment. Expression of *GhNPC6c* and *GhNPC6d* were down-regulated under both low-phosphate and salt treatments, whereas they were up-regulated at most time points under ABA treatments. *GhNPC6a*, *GhNPC6b*, *GhNPC6c*, and *GhNPC6d* were up-regulated at the beginning but then down-regulated under the drought treatment.

## Discussion

Non-specific phospholipases C (NPCs) were discovered as a type of plant phospholipid-cleaving enzyme [1, 2, 4]. The six-gene family was established in Arabidopsis, and accumulating evidence suggests the involvement of Arabidopsis NPCs in abiotic stress responses as well as phytohormone activity [1, 2, 12–19]. To date, however, only a few physiological functions and signaling roles have been clearly illustrated, most of which are limited to Arabidopsis. In recent years, genome-wide identification and expression analysis have been carried out for the NPC gene family in rice and soybean [41, 42], but information of the NPC gene family in other plant species remains largely unknown. Hence, cloning, structural, functional and evolutionary analysis of the non-specific phospholipase C gene family in upland cotton appears important for pursuing further insight into the role of plant NPC in numerous biological processes.

### NPC gene family in *G. hirsutum*

The completion of the sequencing of *G. hirsutum* provides great help for our research work. However, it is difficult to accurately sequence and assemble *G. hirsutum* genome owing to its large and complex allotetraploid genome [43]. So, the simple genome-wide identification based on genome sequencing for such complex allotetraploid *G. hirsutum* is not extremely accurate. There are two versions of the genome sequencing of upland cotton including Gossypium_hirsutum_v1.0 and Gossypium_hirsutum_v1.1. We identified eleven *NPC* genes from Gossypium_hirsutum_v1.1 genome and nine *NPC* genes from Gossypium_hirsutum_v1.0 genome, respectively. The nine *NPC* genes identified from Gossypium_hirsutum_v1.0 genome which were consistent with the result of Zhang et al. have corresponding sequences in that eleven *NPC* genes from Gossypium_hirsutum_v1.1 genome, but the sequences are different [44]. The number of *NPC* genes and the true sequences need to be verified by experiment. In this study, a total of 11 *NPC* genes were cloned in *G. hirsutum*, and five genes were consistent with the Gossypium_hirsutum_v1.1 genomic data, three genes were consistent with the Gossypium_hirsutum_v1.0 genomic data. The other three genes have 1, 3, 4 base differences with *G. hirsutum* genome sequencing results, respectively. The difference between exist *GhNPC* sequences and the *G. hirsutum* genome sequencing results may be due to inaccuracies in sequencing and assemble, or/and due to differences between varieties (Zhong9807 and TM-1). In addition, there are also differences in the expression levels of three corresponding genes (*GhNPC1b*, *GhNPC3b*, *GhNPC6a*) between our results and the paper of Zhang et al. at salt and PEG treatments. The differences in the expression levels of three corresponding genes (*GhNPC1b*, *GhNPC3b*, *GhNPC6a*) between our results and the paper of Zhang et al. may be because the material were taken from different tissues of different varieties at different growth stages. Our material is the root tissue of Zhong9807 at the six-leaf stage, while the material of Zhang et al. is the leaf tissue of TM-1 (the age is not applicable). Anyway, it is not comprehensive to characterize *GhNPC*s only using nine *NPC* genes identified from Gossypium_hirsutum_v1.0 genome.

Gene structure analysis showed that *GhNPC*s had preserved a relatively simple constant exon-intron composition. Most of them have two introns, though three of them (*GhNPC2a*, *GhNPC2b*, and *GhNPC3a*) have three introns (Fig. 2b). A similar phenomenon was observed in Arabidopsis, which contains 2–4 introns, and in rice, which contains 0–3 introns [42]. This suggested that plant *NPC* genes may have a relatively simple constant exon-intron composition. As shown in Fig. 2c, proteins with similar motif compositions were clustered in the same class. Present in all GhNPCs were the motifs 1, 3, 5, 7, and 9. Motif 1, 2, 3, 4, 6, and 9 were annotated as the phosphoesterase. Additional, four motifs (motifs 1, 3, 4, and 9) are likely important for NPC catalytic activity [1]. Thus, motif 1, motif 3, and motif 9 were crucial for NPC catalytic activity. Consistent with the previous reports, GhNPCs were also composed by the beta sheet and several alpha helices [1, 4]. The putative active site residues and the active site-stabilizing ion pairs (aspartate-arginine) of GhNPCs were conserved with AtNPCs (Fig. 3), which suggested the common catalytic reaction mechanism between AtNPCs and GhNPCs [1].

### Functional analysis of the *GhNPC* genes

Increasing empirical evidence suggests that *NPC* genes could respond to abiotic stress and signaling transduction in plant. However, until now, the biological and cellular functions of most plant *NPC* genes remain unknown. Functional annotations showed that *GhNPCs* were involved in glycerophospholipid metabolism, ether lipid metabolism, and biosynthesis of secondary metabolites (Additional file 7: Table S4). The expression profile of a gene is always relative to its function. To investigate the function of the *GhNPC*s, we analyzed their expression profiles in different organs and in response to low-phosphate, salt, drought, and ABA. Results of real-time quantitative PCR demonstrated that *GhNPCs* had tissue-specific expression and were significantly altered under different abiotic stress treatments and ABA treatment.

As shown in Fig. 7, *GhNPC1* and *GhNPC6* were ubiquitously expressed, whereas *GhNPC3a* and *GhNPC3b* were highly expressed in root. Similar results have also been found for *NPC1*,*NPC6* and *NPC3* genes in Arabidopsis, and for which *NPC2* is highly expressed in fertility organs but not in roots [15]. Meanwhile, *GhNPC2a* and *GhNPC2b* were also highly expressed in fertility organs such as petal and ovule. The main difference was that *GhNPC2a* and *GhNPC2b* were also highly expressed in taproot. In addition, *NPC4* in *G. hirsutum* and Arabidopsis also showed different tissue specificity. The differing tissue specificity of *NPC2* and *NPC4* in *G. hirsutum* vs. Arabidopsis suggests that they might have diverse functions in these two species.

Many *GhNPC* genes showed great changes after treatments with low-phosphate, salt, drought, and ABA, which suggests their potential role in regulating upland cotton responses to stress abiotic conditions and in ABA signaling. Previous researcher has reported that Arabidopsis *NPC1* is involved in the plant response to heat [13]. HSE, a *cis*-acting element involved in heat stress responsiveness, was found in the promoter of *GhNPC1a* and *GhNPC1b* (Additional file 6: Excel S1), suggesting that *GhNPC1a* and *GhNPC1b* might also play a role in upland cotton response to heat. Moreover, our results suggest that *GhNPC1a* and *GhNPC1b* were induced by low-phosphate, salt, and drought (Fig. 8), so they likely play roles in cotton response to these three types of stress. The function of the Arabidopsis *NPC1* gene in response to low-phosphate, salt, and drought conditions is still not resolved, and requires further research. Previous studies revealed that Arabidopsis *NPC3* is involved in the BL-mediated signaling in root growth [14]. Our study showed that both *GhNPC3a* and *GhNPC3b* were induced by low-phosphate, drought, and ABA. These results suggest that *NPC3* is involved in regulating abiotic stress responses and phytohormone signaling transduction. In Arabidopsis, *NPC4* plays an important role in responses to ABA and phosphate starvation, drought, and salt [12, 15, 16]. Our data shows that *GhNPC4* was induced by salt, but not induced by drought, which is not completely consistent with Arabidopsis *NPC4*. Arabidopsis *NPC2* and *NPC6* have not been studied for their function yet, but we found that *GhNPC2* and *GhNPC6* were involved in regulating abiotic stress responses and phytohormone signaling transduction. Additionally, *cis*-elements play a significant role in plant stress responses [45], and many *cis*-elements in the promoters of the *GhNPC* genes were related to heat, drought, low-temperature, defense stresses, anaerobic induction, wound-responses, and fungal elicitors, ABA, SA, MeJA, GA, IAA, and ethylene (Fig. 6 and Additional file 6: Excel S1). Taken together, our results suggest that *GhNPCs* are involved in regulating abiotic stress responses and phytohormone signaling transduction, and that some NPCs may have diverse functions in *G. hirsutum* and Arabidopsis. We plan to verify the functional characteristics of the *GhNPC* genes in our future work.

### Evolutionary analyses of the *GhNPC* genes

The phylogenetic analysis suggested that the entire cotton *NPC* gene family could be differentiated into four major classes: NPC1, NPC2, NPC6 and NPC3-5 (Fig. 5). Consistent with a previous report [1], we did not identified the NPC3–5 subfamily in the majority of species that we analyzed. In addition, *P. patens* and *S. moellendorffii* only have NPC1. It is possible that NPC1 subfamily is the ancestral NPC, which generated other NPC types, and some NPC genes had undergone species-specific evolutionary processes.

Plant genomes have undergone several rounds of whole-genome duplication (WGD) [46]. Duplicate genes are the prominent source of new genes and novel functions [47]. The allotetraploid *G. hirsutum* species resulted from hybridization of the two ancestral species *G. arboreum* and *G. raimondii* followed by chromosome doubling about ~1.5 million years ago (MYA) [43, 48–50]. Both *G. arboreum* and *G. raimondii* underwent 2 whole genome duplications during their evolution, one was the paleohexaploidization event common to all eudicots at ~130.8 MYA and the other was cotton-specific whole genome duplication at ~16.6 MYA [43, 50–52]. Syntenic analysis showed that four pairs of *GhNPC* (*GhNPC1a* and *GhNPC1b*, *GhNPC2a* and *GhNPC2b*, *GhNPC6a* and *GhNPC6b*, *GhNPC6c* and *GhNPC6d*) were segmental/WGD duplicates. Three models (pseudogenized, subfunctionalized, and neofunctionalized) have been proposed to explain why duplicated genes retained after WGD [47, 53–57]. The *K*_*a*_/*Ks* ratio of four *G. hirsutum* duplicated gene pairs were less than 1, which suggested that they underwent purifying selection [57]. Thus, these four duplicated genes are subfunctionalized genes and have partitioned their functions between pairs. Various functional roles may be the evolutionary driving force for the retention of these four pairs duplicate genes.

## Conclusion

In this study, we first cloned 11 *NPC* genes in *G. hirsutum* and they were designated as *GhNPC1a*, *GhNPC1b*, *GhNPC2a*, *GhNPC2b*, *GhNPC3a*, *GhNPC3b*, *GhNPC4*, *GhNPC6a*, *GhNPC6b*, *GhNPC6c*, and *GhNPC6d*. These eleven GhNPCs were annotated as phospholipase C, which had typical phosphoesterase domains, had hydrolase activity acting on ester bonds, and were involved in glycerophospholipid metabolism, ether lipid metabolism, and biosynthesis of secondary metabolites. The *GhNPC*s had a relatively simple constant exon-intron composition. Motif 1, motif 3, and motif 9 were crucial for NPC catalytic activity. The backbone of GhNPCs was composed by the beta sheet and several alpha helices. Our results also suggested that *GhNPCs* are involved (1) in regulating key abiotic stress (low-phosphate, salt, and drought) responses and (2) in ABA signaling transduction. In addition, four pairs of *GhNPC*s were generated by whole-genome duplication and they underwent purifying selection. Various functional roles may be the evolutionary driving force for the retention of duplicate genes. Our analysis of the GhNPC gene family broadens our insight into the roles of *NPC* genes in plant abiotic stress responses and signaling transduction, provides the foundation for further functional characterization of the *GhNPC* gene family and for potential applications towards the genetic improvement of cotton.

## Additional files

Additional file 1: Table S1. List of primers used for gene amplification. (DOC 40 kb)

Additional file 2: Data Set S1. The protein sequences used to generate phylogenetic tree. (DOC 54 kb)

Additional file 3: Table S2. List of primers used in quantitative real time-PCR expression analysis (DOC 39 kb)

Additional file 4: Data Set S2. The CDS sequences of *GhNPC*s (DOC 39 kb)

Additional file 5: Table S3. The *K*_*a*_/*Ks* ratio of four *G. hirsutum* duplicated gene pairs (DOC 30 kb)

Additional file 6: Excel S1. Analysis of *cis*-elements in *GhNPC* gene promoters (XLSX 22 kb)

Additional file 7: Table S4. Functional annotations of GhNPCs. Functional annotations of GhNPCs were predicted using the Cotton Functional Genomics Database (DOC 88 kb)

Additional file 8: Table S5. Sequence information of twenty motifs (DOC 38 kb)

## Abbreviations

Aa: amino acid
ABA: abscisic acid
AcpA: Acid Phosphatase A
PC: phosphatidylcholine
*C*_T_: threshold cycle
GA: gibberellin
GSDS: Gene Structure Display Server
IAA: auxin
*K*_*a*_: non-synonymous
*Ks*: synonymous
MeJA: methyl jasmonate
ML: Maximum-likelihood
Mw: molecular weight
MYA: million years ago
NCBI: National Center of Biotechnology Information
NPC: Nonspecific phospholipase C
PE: phosphatidylethanolamine
PFD: photon flux density
pI: isoelectric points
SA: salicylic acid
TAIR: The Arabidopsis Information Resource
WGD: whole-genome duplication
