# Supplementary material for: Structural, expression and evolutionary analysis of the non-specific phospholipase C gene family in Gossypium hirsutum
Source: BMC Genomics. 2017 Dec 19;18:979. doi: 10.1186/s12864-017-4370-6 (PMC5738194; doi:10.1186/s12864-017-4370-6)
Supplement: Supplementary file 1 — List of primers used for gene amplification. (DOC 40 kb) [file 12864_2017_4370_MOESM1_ESM.doc]

**Additional File1: Table S1** List of primers used for gene amplification.

| Primer name | Sequence (5'–3') |
| --- | --- |
| GhNPC1a-1: | GGGGTACCATGGAAAACCGTTCTTTTGA |
| GhNPC1a-2: | CGAGCTCCTAATAGGCTTCCACATGTT |
| GhNPC1b-1: | GGGGTACCATGGAAAACCGTTCTTTTGA |
| GhNPC1b-2: | CGAGCTCCTAATAGGCTTCCACATGTT |
| GhNPC2a-1: | GGGGTACCATGTTCAAACCTGCTAACACT |
| GhNPC2a-2: | CGAGCTCTTATGGATGTTGAGATGAA |
| GhNPC2b-1: | GGGGTACCATGTTCAAACCTGCTAACACT |
| GhNPC2b-2: | CGAGCTCTTATGGATGTTGAGATGAA |
| GhNPC3a-1: | GGTACCATGGCAGTTGAAACAAGCT |
| GhNPC3a-2: | GAGCTCTCAACGATCACAAACCAAA |
| GhNPC3b-1: | GGTACCATGGTTTCATTAATTAATAT |
| GhNPC3b-2: | GAGCTCTCAACGATCACAAACCAAA |
| GhNPC4-2: | GGGGTACCATGTATGTATATATATCTCAACCCATTCCA |
| GhNPC4-1: | CGAGCTCTCAATCATGGCAGACAAGACATTT |
| GhNPC6a-1: | TCTAGAATGGGGGAATCCAAAGCCA |
| GhNPC6a-2: | GGTACCCTAGTTGTGGATCGAAGAA |
| GhNPC6b-1: | TCTAGAATGGGGGAATCCAAAACCA |
| GhNPC6b-2: | GGTACCCTAGTTGTGGATCGAAGAA |
| GhNPC6c-1: | TCTAGAATGGAGGGCTCATTTTCATT |
| GhNPC6c-2: | GAGCTCTTACGGATTCGAAGATCTCGT |
| GhNPC6d-1: | GGTACCATGGAGCGCTCATTTTCCTT |
| GhNPC6d-2: | GAGCTCTTACGGATTCGAAGATCTCGT |
