# Supplementary material for: Structural, expression and evolutionary analysis of the non-specific phospholipase C gene family in Gossypium hirsutum
Source: BMC Genomics. 2017 Dec 19;18:979. doi: 10.1186/s12864-017-4370-6 (PMC5738194; doi:10.1186/s12864-017-4370-6)
Supplement: Supplementary file 2 — The protein sequences used to generate phylogenetic tree. (DOC 54 kb) [file 12864_2017_4370_MOESM2_ESM.doc]

**Additional File2: Data Set S1.** The protein sequences used to generate phylogenetic tree.

>GhNPC1a

MENRSFDHLLGWLKSTRPDIDGLSGTESNPVNVADPNSPFISVSDDALFVDSDPGHSFQAIREQIFGSNDSSADSAPMNGFAQQAESMGEGMGRTVMSGFKPSRLPVYTKLANEFGVFDRWFASVPASTQPNRFYVHSATSFGATSNVKKDLIHGFPQKTIFDSLDENGLSFGIYYQNIPATLFFKSLRKLKFLTKFHNYALKFRLHARLGKLPNYVVVEQRYFDVKEFPANDDHPSHDVARGQRFVKEVYEILRSSPQWKEMALLITYDEHGGFYDHVPTPVSGVPNPDGIVGPDPFYFKFNRLGVRVPTLLVSPWIDKATVIHEPTGPTPSSQFEHSSIPATVKKLFNLNSNFLTKRDAWAATFENYFKLRTTPRTDCPETLPEVTTSLRPWGPKEDASLSEFQVELVQLASQLNGDYVLNTYPYIGKSMRVGEANRYVEDAVKRFLEAGKAAIRAGANESAIVTMRPSLTSRIEDRGQHVEAY

>GhNPC1b

MENRSFDHLLGWLKSTRPDIDGLSGTESNPVNVADPNSPFISVSDDALFVDSDPGHSFQAIREQIFGSNDSSADSAPMNGFAQQAESMGEGMGRTVMSGFKPSRLPVYTKLANEFGVLDRWFASVPASTQPNRFYVHSATSFGATSNVKKDLIHGFPQKTIFDSLDENGLSFGIYYQNIPATLFFKSLRKLKFLTKFHNYALKFRLHARLGKLPNYVVVEQRYFDVKEFPANDDHPSHDVARGQRFVKEVYEILRSSPQWKEMALLITYDEHGGFYDHVPTPVSGVPNPDGIIGPDPFYFKFNRLGVRVPTLLVSRWIDKATVIHEPTGPTPSSQFEHSSIPATVKKLFNLNSNFLTKRDAWAATFENYFKLRTTPRTDCPETLPEVTTSLRPWGPQEDASLSEFQVELVQLASQLNGDYVLNTYPSIGKSMRVGEANRYVEDAVKRFLEAGKAAIRAGANESAIVTMRPSLTSRIEDRSQHVEAY

>GhNPC2a

MFKPANTAIFFFFVLFNSFSCHGGPVKTIVVLVMENRSFDHMLGWMKKINPQINGVDGTEWNPLSTTDPNSKKLFFQNQAQFVDPDPGHSFQAIREQIFGSNDTSANPPPMNGFAQQAYSMDLSTTMSQNVMNGFDPEMVAVYKSLVSEFAVFDRWFASVPSSTQPNRLYVHSATSAGATSNIPALLVKGYPQRTIFENLDDAGISWGIYYQNIPATLFYKNLRKLKYLFRFRPYGVTFKKHAQEGKLPGYVVVEQRYMDTKLEPANDDHPSHDVYQGQMFVKEVYETLRASPQWNQTLLIITYDEHGGFYDHVATPVTGVPSPDGIVGPEPFFFHFDRLGVRVPTIMVSPWIDKGTVVHGANGRPFPTSEFEHSSIPATVKLLFNLTSPFLTKRDEWAATFESILRTRSDPRTDCPETLPTPARIRRGEAIEEAKLSEFQQELVQLAAVLKGDHILTSYPERIGKDMSVKEGKEYMEDAVKRFFEAGHYAKKMGVDGEQIVQMKPSLTTRSSKPSSQHP

>GhNPC2b

MFKPASTAIFFFFFSCHGGPIKTIVVLVMENRSFDHMLGWMKKINPEINGVDGTEWNPLSTTDPNSKKLFFQNQAQFVDPDPGHSFQAIREQIFGSNDTSTNPPPMNGFAQQAYSMDPSTTMSQNVMNGFDPEMVPVYKSLVSEFAVFDRWFASVPSSTQPNRLYVHSATSAGATSNIPALLVKGYPQRTIFENLDAAGISWGIYYQNIPATLFYKNLRKLKYLFRFRPYGVTFKKHAQEGKLPGYVVVEQRYMDTKLEPANDDHPSHDVYQGQMFVKEVYETLRASPQWNQTLLIITYDEHGGFYDHVATPVTGVPSPDGIVGPEPFFFHFDRLGVRVPTIMVSSWIDKGTVVHGANGRPFPTSEFEHSSIPATVKLLFNLTSPFLTKRDEWAATFESILRTRSDPRTDCPETLPTPARIRRGEANEEAKPSEFQQELVQLAAVLKGDYILTSYPERIGKEMSVKEGKEYMEDAVKRFFEAGHFAKKMGVDGEHIVQMKPSLTTRSSKPSSQHP

>GhNPC3a

MAVETSSATPSPVKTVVVLVQENRSFDHMLGWFKTINPEIDGVTGSESNPISTSDPNSTQITFKDTAGYVDPDPDHSFQAIYEQVSGKTWDTSNPDPNPGIKMNGFVQNAERTTPGLSETVMNGFKPEAVPVFKQLVTEFAVCDRWFASLPASTQPNRLYVHSATSHGAMSNNTQQLIEGFPQKTIFESLEENGYSFGIYYQSFPSTLFYRKLRHLKYVDNFHQYDLSFKRHCKDGKLPNYVVIEPRYFDILTAAANDDHPSHDVSEGQKLVKEIYEALRSSPQWNEILFLVIYDEHGGFYDHVPTPTGVPSPDDIVGPEPYNFKFDRLGCRVPAIMVSPWIEPGTVLHRPSGPDPTSEFEHSSIAATLKKIFNLKEFLTKRDAWAGSFDIVVNRSTPRTDCPEKLAEPVKMRDSDAKETAKLSDFQEELVQAAAALKGDPFNLVENMTVSSGLKYVEDAFKKFYDDGQKAKEINEVEDTVSADASTRRTTASKTFMQKVFSCLVCDR

>GhNPC3b

MVSLINIRKLRHLKYVDNFHQYDLSFKRHCKDGKLPNYVVIEPRYFDILTAAANEDHPSHDVSEGQKLVKEIYEALRSSPQWNEILFLVIYDEHGGFYDHVPTPTGVPSPDDIVGPEPYNFKFDRLGCRVPAIMVSPWIEPGTVLHRPSGPDPTSEFEHSSIAATLKKIFNLKEFLTKRDAWAGSFDIVVNRSTPRTDCPEKLADPVKMRDTDAKETAKLRDFQEELVQAAAALKGDPFNLVENMTVSSGLKYVEDAFKKFYDDGQKAKEINEVEDTVSADASTRRTTASKTFMQKVFSCLVCDR

>GhNPC4

MYVYISQPIPNQQRTYKNRTMVSQGSNSASSYPIKTIVILVQENRSFDHMLGWFKSLNPEIDGVTGSESNPISTSDPNSPMVFFKDNSEYVDPDPAHSIQAIYEQVFGHPWSSDLPNPPHEPTMNGFAQNAERTEKGMAEAVMKGFKPDAVPVYKELASKFGICDRWFASVPASTQPNRMFVHSATSYGQESNDAIKLIKGFPQKTIFESLDESGFSFGIYYQYPPSTLFFRNLRQMKYLKNFHQFDLHFKKHCEEGKLPNYVVVEQRYFDLLSVPANDDHPSHDVSEGQKFVKQVYEALRSSPQWKEMLLVITYDEHGGFYDHVPTPTNGVPSPDDIVGPEPYHFKFDRLGVRVPTFFVSPWIEPGTGKSRESPLLLSFNLGYLDHVCMVMEFAVIHRPLGPYPTSQFEHSSIPATVKKIFNLKEFLTKRDAWAATFEGVINRKNPRVDCPVTLPEPVKMRPTEAKETAKLSDFQKELVQMAAVLNGDHKSDMYPHKLVEKMTVAEAAKYVNGAFNKFCDECQRGGIHESEIVELGKQVERPKGRSFIYKFFKCLVCHD

>GhNPC6a

MGESKASPPPSFSFIFSLFLTVACLFTPLGAQQQSPIKTIVVLVMENRSFDHMLGWMKQHVNPSINGVTGDECNPVSTKNPNPESICFTDDAEFVDPDPGHSFEAVEQQVFGSSTIPSMSGFVEQALSMSKNLSETVMKGFRPESVPVYAALVKEFAVFDRWFSSIPGPTQPNRLFVYSATSHGSTSHVKKQLAHGYPQKTIFDSLHENDKDFGVYFQNIPTTLFYRNLRKLKYVFKFHQFDLKFKKDARKGKLPSLTVIEPRYFDLKGLPANDDHPSHDVANGQKLVKEVYEILRASPQWNQTLLVITYDEHGGFYDHVHTPYINVPSPDGNTGPAPSFFKFDRLGVRVPTIMVSPWIKKGTVISGPKGPFPNSEFEHSSIPATIKKMFNLSSNFLTHRDAWAGTFERVVGELSSPRTDCPEKLPEAAPLRTTAANEDAGLSEFQSEVVQLASVLNGDHFLSGFAEEMHTKMSVKGAHEYVKGAVSRFIRASKEAIKLGADESAIVDMRSSLTTRSSSIHN

>GhNPC6b

MGESKTSPPPSFSFIFSLFLTVACLFTPLGAQQQSPIKTIVVLVMENRSFDHMLGWMKQHVNPSINGVTGDECNPVSTKNPNPESICFTDDAEFVDPDPGHSFEAVEQQVFGSSTIPSMSGFVEQALSMSKNLSETVMKGFRPESVPVYAALMKEFAVFDRWFSSIPGPTQPNRLFVYSATSHGSTSHVKKQLAHGYPQKTIFDSLHENGKDFGVYFQNIPTTLFYRSLRKLKYVFKFHQFDLKFKKDARKGKLPSLTVIEPRYFDLKGLPANDDHPSHDVANGQKLVKEVYEILRASPQWNQTLLVITYDEHGGFYDHVHTPYINVPSPDGNTGPAPSFFKFDRLGVRVPTIMVSPWIKKGTVISGPKGPFPNSEFEHLSIPATIKKMFNLSSNFLTHRDAWAGTFEHVVGELSSPRTDCPEKLPEAALLRTTEANEDAGLSEFQSEVVQLASVLNGDHFLSSFAEEMRTKMSVKGAHEYVKGAVSRFIRASKEAIKLGANESTIVDMRSSLTTRSSSIHN

>GhNPC6c

MEGSFSFIFLLFLLPFVVSQGSPIKTIVVLVMENRSFDHMVGWMKQSINPTINGVTGNECNPISTKTPNPKSICFTNDAQFVDPDPGHSFEAVEQQVFGSTLSSFPSMSGFVEQAFSISPNMSETVMKGFKPEAVPIYATLVKEFAVFDRWFSSIPGPTQPNRLFVYSATSHGSTSHVKKQLAQGYPQKTIFDSLHENGKDFGVYFQNIPTTLFYRNLRKLKYVFKFHQFDLKFKKDALNGKLPSLSVIEPRYFDLKGLPANDDHPSHDVANGQKLVKEVYETLRASPQWNETLLVITYDEHGGFYDHVKTPFVNVPNPDGNTGPAPSFFKFDRLGVRVPTIMVSPWIKKGTVISGPKGPTPNSEFEHSSIPATIKKIFNLSSNFLTHRDAWAGTFEDVVSHLTSPRTDCPETLPDVVPLRATEAKEDAALSEFQSEVVQLAAVLNGDHFLSSFPDEMSKKMTVKEAHEYTKGAISRFIRASKEALKLGAAESAIVDMRSSLTTRSSNP

>GhNPC6d

MERSFSFIFLLFILPFVVSQESPIKTIVVLVMENRSFDHMVGWMKQGINPTINGVTGNECNPISTKTPNPKSICFTNDAQFVDPDPGHSFEAVEQQVFGSTPSSFPSMSGFVEQAFSISPNMSETVMKGFRPEAVPIYASLVKEFAVFDRWFSSIPGPTQPNRLFVYSATSHGSTSHVKKQLAQGYPQKTIFDSLHENGKDFGVYFQNIPTTLFYRNLRKLKYVFKFHQFDLKFKKDALNGKLPSLSVIEPRYFDLKGLPANDDHPSHDVANGQKLVKEVYETLRASPQWNETLLVITYDEHGGFYDHVKTPFVNVPNPDGNTGPAPSFFKFDRLGVRVPTIMVSPWIKKGTVISGPKGPTPNSEFEHSSIPATIKKIFNLSSNFLTHRDAWAGTFEDVVSHLTSPRTDCPETLPDVVPLRTTEAKEDAALSEFQSEVVQLAAVLNGDHFLSSFPDEMSKKMTVKEAHEYTKGAVSRFIRASKEALKLGAAESAIVDMRSSLTTRSSNP

>AtNPC1

MAFRRVLTTVILFCYLLISSQSIEFKNSQKPHKIQGPIKTIVVVVMENRSFDHILGWLKSTRPEIDGLTGKESNPLNVSDPNSKKIFVSDDAVFVDMDPGHSFQAIREQIFGSNDTSGDPKMNGFAQQSESMEPGMAKNVMSGFKPEVLPVYTELANEFGVFDRWFASVPTSTQPNRFYVHSATSHGCSSNVKKDLVKGFPQKTIFDSLDENGLSFGIYYQNIPATFFFKSLRRLKHLVKFHSYALKFKLDAKLGKLPNYSVVEQRYFDIDLFPANDDHPSHDVAAGQRFVKEVYETLRSSPQWKEMALLITYDEHGGFYDHVPTPVKGVPNPDGIIGPDPFYFGFDRLGVRVPTFLISPWIEKGTVIHEPEGPTPHSQFEHSSIPATVKKLFNLKSHFLTKRDAWAGTFEKYFRIRDSPRQDCPEKLPEVKLSLRPWGAKEDSKLSEFQVELIQLASQLVGDHLLNSYPDIGKNMTVSEGNKYAEDAVQKFLEAGMAALEAGADENTIVTMRPSLTTRTSPSEGTNKYIGSY

>AtNPC2

MSIKAFALIQLLSVTILYNHVHATSPIKTIVVVVMENRSFDHMLGWMKKLNPEINGVDGSESNPVSVSDPSSRKIKFGSGSHYVDPDPGHSFQAIREQVFGSNDTSMDPPPMNGFVQQAYSEDPSGNMSASVMNGFEPDKVPVYKSLVSEFAVFDRWFASVPSSTQPNRMFVHSGTSAGATSNNPISLAKGYPQRTIFDNLDDEEFSFGIYYQNIPAVLFYQSLRKLKYVFKFHSYGNSFKDHAKQGKLPAYTVIEQRYMDTLLEPASDDHPSHDVYQGQKFIKEVYETLRASPQWNETLLIITYDEHGGYFDHVPTPVRNVPSPDGIVGPDPFLFQFNRLGIRVPTIAVSPWIEKGTVVHGPNGSPFPSSEYEHSSIPATVKKLFNLSSPFLTKRDEWAGTFENILQIRKEPRTDCPETLPEPVKIRMGEANEKALLTEFQQELVQLAAVLKGDNMLTTFPKEISKGMTVIEGKRYMEDAMKRFLEAGRMALSMGANKEELVHMKTSLTGRRP

>AtNPC3

MVEETSSGGGSSASPIKTIVVLVQENRSFDHMLGWFKELNPEIDGVSESEPRSNPLSTSDPNSAQIFFGKESQNIDPDPGHSFQAIYEQVFGKPFSDESPYPDPKMNGFVQNAEAITKGMSEKVVMQGFPPEKLPVFKELVQEFAVCDRWFSSLPSSTQPNRLYVHAATSNGAFSNDTNTLVRGFPQRTVFESLEESGFTFGIYYQSFPNCLFYRNMRKLKYVDNFHQYHLSFKRHCKEGKLPNYVVIEPRYFKILSAPANDDHPKNDVVEGQNLVKEIYEALRASPQWNEILFVVVYDEHGGYYDHVPTPVIGVPNPDGLVGPEPYNFKFDRLGVRVPALLISPWIEPGTVLHEPNGPEPTSQFEHSSIPATLKKIFNLKSFLTKRDEWAGTLDAVINRTSPRTDCPVTLPELPRARDIDIGTQEEDEDLTDFQIELIQAAAVLKGDHIKDIYPFKLADKMKVLDAARYVEEAFTRFHGESKKAKEEGRDEHEIVDLSKGSTRHSTPKSFVQKLFSCLICDN

>AtNPC4

MIETTKGGSGSYPIKTIVVLVQENRSFDHTLGWFKELNREIDGVTKSDPKSNTVSSSDTNSLRVVFGDQSQYVNPDPGHSIQDIYEQVFGKPWDSGKPDPNPGHPNMSGFAQNAERNKKGMSSAVMNGFKPNALPVYKELVQNFAICDRWFASVPASTQPNRLYVHSATSHGATSNDKKLLLEGFPQKTIFESLDEAGFSFGIYYQFPPSTLFYRNLRKLKYLTHFHQYGIQFKKDCKEGKLPNYVVVEQRWFDLLSTPANDDHPSHDVSEGQKLVKEVYEALRSSPQWNEILFIITYDEHGGFYDHVPTPVDGVPNPDGILGPPPYNFEFNRLGVRVPTFFISPWIEPGTVIHGPNGPYPRSQYEHSSIPATVKTIFKLKDFLSKRDSWAGTFESVITRDSPRQDCPETLSTPIKLRGTMAKENAQLSEFQEDLVIMAAGLKGDYKNEELIHKLCKETCVADASKYVTNAFEKFLEESRKARDRGCDENDIVYCVDDDDDHVVIPPQSHSEASNAAAQPKTQTSFFNKLFSCFIRHD

>AtNPC5

MAETKKGSESYPIKTIVVLVQENRSFDHTLGWFKELNREIDGVMKSDQKFNPGFSSDLNSHNVVFGDQSQYVDPNPGHSIRDIYEQVFGKPWDSGHPDPNPGPATMSGFAQNAERKMKGMSSAVMNGFKPDALPVYKELVQNFAICDRWFASVPGATQPNRLFIHSATSHGTTNNERKLLIEGFPQKTIFESLDEAGFTFGIYYQCFPTTLFYRNLRKLKYLTRFHDYGLQFKKDCKEGNLPNYVVVEQRWYDLLLNPANDDHPSHDVSEGQKLVKEVYEALRSSPQWNEILFIITYDEHGGFYDHVPTPLDGVPNPDGILGPPPYNFEFNRLGVRVPTFFISPWIEPGTVLHGSNGPYLMSQYEHSSIPATVKKIFKLKDFLTKRDSWAGTFESVITRNSPRQDCPETLSNPVKMRGTVAKENAELSDFQEELVIVAAGLKGDYKNEELLYKLCKKTCVSDASKYVTKAFDKFVEESKKARERGGDENDIVFCVDDDDDHNVVKPPPSQSEPSHATPWSN

>AtNPC6

MKPSSASRFSLTFSHFLTLYCLLTQTHVAQGSHQWQSPIKTVVVLVLENRSFDHLLGWMKNSVNPTINGVTGQECNPVPNSTQTICFTSDAEFVDPDPGHSFEAVEQQVFGSGPGQIPSMMGFVEQALSMPGNLSETVMKGFRPEAVPVYAELVKEFAVFDRWFSSIPGPTQPNRLFVYSATSHGSTSHVKKQLAQGYPQKTIFDSLHSNDIDFGIYFQNIPTTLFYRNLRQLKYIFNLHQYDLKFKKDAAKGKLPSLTVIEPRYFDLKGLPANDDHPSHDVANGQKLVKEVYEALRSSPQWNETLLVITYDEHGGFYDHVKTPYVGIPNPDGNTGPAPGFFKFDRLGVRVPTIMVSPWIQKGTVVSEAKGPTESSEYEHSSIPATIKKLFNLSSNFLTHRDAWAATFEDVVSHLTTPRTDCPMTLPEVAPMRATEPKEDAALSEFQGEVVQLAAVLNGDHFLSSFPEEIGKKMTVKQAHEYVKGATSRFIRASKEAMKLGADKSAIVDMRSSLTTRPHN

>GmNPC1a

MPLRRRVPLPLLLLLLLLSPAATSAVAFRKKHKIPGPIKTIVVIVMENRSFDHVLGWLKSSRPDIDGLTGTESNPLSVSSRSSPTVPVSDDALFIDSDPGHSFQAIREQIFGSNDTSAVPPPMNGFAQQAESILPGMSKTVMSGFKPQTLPVYTALANQFGLFDKWFASVPASTQPNRFYVHSATSHGAMSNVRKDLIHGFPQKTIFDSLNENNLSFGIYYQDISATLFFKSLRKLKNAVKFHDYALKFKKHAEKGKLPNYVVVEQRYFDVEVFPANDDHPSHDVAAGQMFVKEVYEVLRKSPQWEEMAVLITYDEHGGFYDHVATPVEGVPNPDGIIGPHPYYFGFDRLGVRVPTFIISPWIDKGTVIHEAEGPTPYSQYEHSSIPATVKKLFNLKSNFLTKRDAWAGTFEKYFYIRDTPRDDCPETLPDIKMLRQHGPREDSSLSEFQVELIQLASQLNGDYVLNSYPNIGKTMTVKEANRYAEDAVKRFLEAAKAALKAGANESAIVTMRPSLTSRVADGDNHKLVESY

>GmNPC1b

MSLRRGVPLSLLVLFLLLVSPAATLAFRKKHKIPGPIKTIVVIVMENRSFDHVLGWLKSSRPDIDGLTGSESNPLSVSSPSSATIPVTDDALFIDADPGHSFQAIREQIFGSNDTSAVPPPMNGFAQQAESILLGMSKTVMSGFKPHTLPVYTALANQFGLFDKWFASVPASTQPNRFYIHSATSHGAMSNVRKDLIHGFPQKTIFDSLNENGLSFGVYYQNIPATLFFKSLRKLKNAVKFHDYALKFKKHAEKGKLPNYVVVEQRYFDVEVFPANDDHPSHDVAAGQMFVKEVYEVLRKSPQWEEMAVLITYDEHGGFYDHVATPVEGVPNPDGIVGPHPYYFRFDRLGVRVPTFIISPWIDKGTVIHEAEGPTPYSQYEHSSIPATVKKLFNLKSNFLTKRDAWAGTFEKYFYIRDTPRDDCPETLPDIKMLRQHGPREDSSLSEFQVELIQLASQLNGDYVLNSYPNIGKTMTVKEANRYAEDAVKRFLEAAKAALKAGANESAIVTMRPSLTSRVAEGDHHKLVESY

>GmNPC2

MATQRSHHSPILFSSLILTLFVLYFPRCHHAIPNNPIKTVVVLVMENRSFDHMLGWMKRLNPAIDGVTGSESNPLSVSDPDSKRFFFRDRAHFVDPDPGHSFQAIREQIFGSNDSSLDPPPMNGFVQQAYSMDNTSHMSENVMNGFDPDLVAVYKTLVSEFAVFDRWFASVPASTQPNRLFVHSATSGGATSNVAAKLTAGYPQQTIFDSLHDAGHDFGIYYQNIPATLFYRNLRKLKYVLKFHIYDVSFKQHAKEGKLPSYTVVEQRYMDTKLLPANDDHPSHDVYEGQVFVKEVYETLRASPQWNETLFLITYDEHGGFYDHVPTPARGVPSPDGIVGPEPFNFTFNRLGVRVPTIAISPWIEKGTVVHGPNGSPSPTSEYEHSSIPATVKKLFNLPSFLTNRDAWAGTFEGIVQTRTEPRTDCPEKLPTPEKIRKGEPNEDAKLSEFQQELIQLAAVIKGDNILTSFPGTIGKDMTVKQGKYYMDDAVRSFFEAGRYARKMGVNEEHIVQMKPSLTTRSSKSPNTNP

>GmNPC4a

MASNSSTNNAGYPIKTIVVLVQENRSFDHMLGWMKSLDPKINGITGSESNPISTSNPNSNLVQFSDQSVYVDPDPGHSIQDIYEQIFGEPWSEASTAKKLPPTMQGFAQNAGRQAVPKNATATMMETVMNGFKPDLIPVYKELVKEYAVCDCWFASVPASTQPNRLYVHSATSHGLTSNDTNKLIGGLPQKTIFDSLDENGFSFGIYYQSPPATLFYRNLRKLKYVDNFRPFDLFKKHCKEGKLPNYVVIEQRFFDLLSIPGNDDHPSHDVSEGQKFVKEVYEALRGSPQWNETLFVIVYDEHGGFYDHVPTPVEGVPSPDDIVGPEPFKFQFDRLGVRIPAIIVSPWIEPGTVLHGPSGPSPTSQYEHSSIPATVKKIFNLPEFLTKRDAWAGTFEGLLTRSSPRTDCPVKLPEPVKLREAPAQEKAKLSEFQEELVQMAATLNGDHRKSIYPDKLTENMSVPDAVKYVEDAFNTFLNECEKAKQNGADESEIVDCADGCSSAPPDSKNFFYNVLSCITCNR

>GmNPC4b

MSSSSGTSATPYPIKTIVVLVQENRSFDHMLGWMKSLNREIDGVTGLESNQVSTFDPNSNRVYFGDQSGFEEPDPGHTVEDVYEQVFGEPWSESSAANKLSPRMKGFAQNSAKQKKGSTAETVMNGYKPDLLPVYKELVKEFAVCDRWFASVPGPTQPNRLYVHSATSHGLTTQDTKKLIGGLPQKTIFDSLDENGFSFGIYYQYPPSTLFFRNLRKLKYIDNFHQFDLKFKKQCKEGKLPNYVVIEQRYFDLLSLPANDDHPSHDVAEGQKFVKEVYEALRASPQWNEMLFVIIYDEHGGFYDHVPTPVDGVPSPDDIAGPEPFKFQFDRLGVRVPTIIISPWIEAGKVLHEPSGPFPTSQYEHSSIPATVKKIFNLPQFLTKRDAWAGTLEDLLSLSTPRTDCPVKLPDPVKLREAASAEQQTQLSEFQEDLIYMAATLNGDHNKSIYHKLTENLTVSEAVKYCEDAFGTFLNECEKAKQSNRIDGSEIVYCARPHTAPQSKNFWHKMLSCILCN

>GmNPC6a

MGSSKPKSSILMFVVFLCVFATAQRQQPIKTVVVLVMENRSFDHMLGWMKESINTLINGVTGDECNPVSTKSPRKDSICFTDDAEFVDPDPGHSFEDVLQQVFGSGSGSIPSMNGFVEQALSMSPNLSETVMKGFKPDSVPIYAALVKEFAVFDRWFSSIPGPTQPNRLFVYSATSHGSTSHIKRQLAKGYPQKTIFDSLHENGLDFGIYFQNIPTTLFYRNLRKLKYIWKFHQYDLKFKRDARDGKLPPLTVIEPRYFDLKGIPANDDHPSHDVAHGQMLVKEVYEALRASPQWNETLFIITYDEHGGFFDHVKTPFVNIPNPDGNTGPAPYFFKFDRLGVRVPTIMVSPWIKKGTVISGAKGPAENSEFEHSSIPATIKKMFNLSANFLTHRDAWAGTFEHVVGDLSSPRTDCPVTLPDVTPLRSTEAKENAGLSEFQSEVVQLAAVLNGDHFLSSFPDEMSKKMSVKEAHEYVRGAVSRFIRASKEAIKLGADESAIVDMRSSLTTRSSVHN

>GmNPC6b

MGSSKPRSFILLLFVFLCVFATAQEQQQPIKTVVVLVMENRSFDHMLGWMKESINTLINGVTGDECNPVSTKSPRKDSICFTDDAEFVDPDPGHSFEDVLQQVFGSSSGSGSIPSMNGFVEQALSMSSPNLSETVMKGFKPDSVPVYAALVKEFAVFDRWFSSIPGPTQPNRLFVYSATSHGSTSHIKRQLAKGYPQKTIFDSMHENGLDFGIYFQNIPTTLFYRNLRKLKYIWKFHQYDLKFKRDARDGKLPPLTVIEPRYFDLKGIPANDDHPSHDVAHGQMLVKEVYEALRASPQWNETLFVITYDEHGGFFDHVKTPFVNIPNPDGNTGPAPYFFKFDRLGVRVPTIMVSPWIKKGTVISGAKGPAENSEFEHSSIPATIKMIFNLSSNFLTHRDAWAGTFEHVVGELSSPRTDCPVTMPDVTPLRSTEAKENAGLSEFQREVVQLAAVLNGDHFLSSFPDEMSKKMSVKEAHEYVRGAVSRFIRASKEAIKLGADESAIVDMRSSLTTRSSVHN

>OsNPC1

MAGGGGRERRGGGRLLVGVLLLTLVVSGHCLESTHHRGLKRRRRKHEIHSPIKTVVVVVMENRSFDHILGWLSRTRPDIDGLNGTQSNRLNASDPSSPEIFVTDEAGYVDSDPGHGFEDIREQIFGSADTSAVPAPMSGFAQNARGMGLGMPQNVMSGFKPESVPVYAALADEFAVFDRWFASVPTSTQPNRLYVHSATSHGLTFNARKDLIHGFPQKTIFDSLEENGLSFGIYYQNIPATLFYQSLRRLKHLVKFHQYSLKFKLHAKWGKLPNYAVIEQRYFDCEMFPANDDHPSHDVARGQRFVKEVYETLRASPQWNETALIITYDEHGGFYDHVPTPVVGVPQPDGIVGPDPYYFKFDRLGVRVPSFLISPWIEKRTVIHEPNGPQDSSQYEHSSIPATVKKLFNLHSNFLTKRDAWAGTFENYFKIRKTPRTDCPEKLPEVTKSLQPFGPKEDSSLSEFQVELIQLASQLNGDHVLNTYPDIGRTMTVGEANRYAEDAVARFLEAGRIALRAGANESALVTMRPALTSRASPSSDLSSEL

>OsNPC2

MAVRRRRPGPVAAAVLLLLAVATQAAASPIKTVVVVVMENRSFDHMLGWMKRLNPEIDGVTGGEWNPTNASDPSSGRVYFGEGAEYVDPDPGHSFQEIRQQIFGSDDASGPARMDGFVQQARSLGDNMTAAVMNGFSPDSVAVYRELVGEFAVFDRWFASVPSSTQPNRLFVHSATSGGATSNNPELLAKGYPQRTIFDNVHDAGLSFGVYYQDVPAVLFYRNLRKLKYLTKFHPFHGAFRDHAARGSLPNYAVVEQHYMDSKSHPANDDHPSHDVFQGQMLVKEVYETLRASPQWNQTLMVVTYDEHGGFYDHVPTPVTGVPSPDGIVGPPPYNFAFDRLGVRVPAIVISPWINKGTVVHGPNGSPTATSEYEHSSIPATVKKLFDLPQDFLTKRDAWAGTFESVVQGRTEPRTDCPEQLPMPMRIRLTEANEEAKLSEFQQELVQLASVLNGDHQLSSLQDTIRDRMNVREGIAYMRGAVKRFFETGMSAKRMGVDDEQIVKMRPSLTTRTSPAIEQP

>OsNPC3

MAGKIKTVVVLVQENRSFDHMLGWMKSLNPEIDGVTGAEFNRATAGDPSSPAIHFGDGSGYVDPDPGHSFQAIYEQVYGDAYTWGTTSPATKPGVPSPPMSGFAQEAEKERAGMSSTVMNGFRPEKVPVYRELVREFAVCDRWFASVPTSTQPNRMFVHSATSHGLVSNDGKQLRAGLPQRTIFDALHDAGHSFGVYYQFPPSVLFYRNMRQLKYVGNFHPYDTAFKRDCKAGKLPNYVVIEQRYFDLKLLPGNDDHPSHDVAHGQRLVKDVYEALRSSPQWHEILFVITYDEHGGFFDHVPTPVAGVPSPDGIVSAAPVSFAFDRLGVRVPTLLVSPWIEPGTVVHDPASCGGAPEPTSQFEHSSIPATVKRIFGLKEFLTRRDAWAGTFDTVLTRAAPREDCPATLPEPPRLRAAEAEEHREVSEFQAELVQLGAALNGDHDGEGYDPEVFVRGMTVAGAAQYCRDAFDRFREECHRCRDGGMDGSHVPMLQPASASASSSAPAADPPATAPAPATPSALSKLCGCFPCFNAS

>OsNPC4

MAAAAAGGKIKTVVVLVMENRSFDHMLGWMKSLNPEIDGVTGDEINHLDAADPTSRAIRFGDGAEYVDPDPGHSMQAIYEQVYGTPFVDARATPITPPGVPSPPMAGFAQQAEKEKPGMADTVMNGFRPEAVPVYRELVRQFAVCDRWFASNPASTQPNRLFVHSATSHGLVSNDTKLLVAGLPQRTIFDSLHDAGFSFGIYYQYPPSTLFYRSLRQLKYAGNFHPFDLAFRRHCAEGKLPNYVVVEQRYFDLKMLPGNDDHPSHDVSEGQRFVKEVYEALRGGPQWEEALLVVTYDEHGGFYDHVPTPVDVPSPDGIVSAAPFFFEFNRLGVRVPALFISPWIEPGTVVHRPSGPYPTSEFEHSSIPATVKKLFNLKSFLTNRDAWAGTFDVVLTRDAPRTDCPATLPEPVKMRPATEAAEQAALTEFQEELVQLGAVLNGDHADEDVYPRKLVEGMTVAEAASYCNAAFKAWMDECDRCRKCGEDGSHIPTVVKPPPPPSTSSSGSSSFASKLLSCFACGRPNKN

>OsNPC6

MWPLSPHRRHLQQHGHSGAMGRRLLLLFLMLAQAPNSNGDSKIKNVVVLALENRSFDHMLGWMQRLLGLPIDGLTGAECNPAPGPGPADSLLHCVSPDADLVVPDDPAHAFEDVLEQLLGFRPNDSTGAAASPSDMSGFVRSAVSVSALLTDAVMRGFTPSRLPAFSALASSFAVFDRWFSSIPGPTQPNRLFLYSATSHGAVAHDKWNLLRGYPQRTIFDSLAADALDYRVYFKTIPTTLFYRRLRTVANAARGTFRRYDAAFRDHARRGLLPALSVIEPRYFDLTGTPADDDHPAHDVANGQRLVKDVYEALRAGPQWNHTLLIITYDEHGGFYDHVPPPNVGVPSPDAIRGPLPFFFRFDRLGVRVPTIMVSPWIRKGTVVGRPPGGPTPTSEYEHSSIPATIKKIFNLSSDFLTRRDAWAGTFEHLFTDLDEPRTDCPETLPEIPPPSSSSSSTKKEDGGWLSDFQRELVQLAAFLNGDYMLSSFAQEYESRMTMTVKQADAYVRRAVKSFLEASKRAKRLGANDSAIVTMRPSLTTATTCCP

>PpNPC1a

MKPPSRTRCGLVYLLFILSLALAPSIAKKARIDGPIKVVVVMVMENRSFDHMLGWLKTLNPEIDGLTGKECNPKNTSNPDSELVCVSNIAEFVDPDPGHSFQAIREQIFGKNETSAIPPPMNGFAQQAESMAKGFSKTVMSGFRPEVVPAYKALAAEYAVFDRWFASAPTSTQPNRFYVHSATSYGAMSNVREELIEGFPQKTIFESIVNAGHTVGIYYQNLPATLFFRNLRKLKFVNKFHDYTLKFRNHARRGVLPNYVVVEQRYFDTKVLPANDDHPSHDVSEGQGFVKEVYEILRASPQWNEMLFIITYDEHGGFYDHVPTPVTNVPNPDGLIGPPPEYFNFRRLGVRVPTLMISPWINKGVVVHGPHGPTADSQYEHSSIPATVRKIFNLPDDFLTARDEWAGTFEHVFAQRKSPRIDCPKQIPSPPWSLRHSPPNESAPLTEFQEELIQLASQLNGDHQHPEYPHLGKRMNVGQAYEYATKAVAKFIETGKAALKAGADPETVIMVSPGVIEMTEDSDNHHESL

>PpNPC1b

MGPIKTIVLLVMENRSFDHFMGLMKKINPEIDGLTGTEDNPITPGDPKAPRIKVSDMAEFVDPDPGHEFEQIAEQIYGSMERVNLTTATMDGFVAQAESVMPGLSKRVMSAFRPEVVPVTTALAMNFAVFDRWFSSVPSSTQPNRLFVHSTTSNGLLSNNEVILLKGMPQRTIYEDVDDAGLSFGVYYQQIPATLFFRNMRKLKYVKNFNTYDRFKSDAKSGKLPNLVVVEQRYFDVAGTPANDDHPTHDISQGQKLIKEVYETLRVSPQWNQILFLITYDEHGGFYDHVPPPAHGVPSPDGVKGPAPHYFNFNRLGVRVPTIAVSPWIEKGTVEHRPQGPTLTSEYEHSSIAATVRTLFSLPQPHLTAREAWAGNFAHIISRTTPRTDTPVTLPSPPWSLRHSHANESRALSLFQEELLLLAKSLRRKLGMGDTANEKSQDQTSALNIGEANFYIQDAVSSFMRRGKAQLQAGLDPNSQVHP

>PpNPC1c

MDRKKAFILLCLQLLLWWTTTARSTPSGPIKTIVLLVMENRSFDHFMGLMKKTNPSIDGLTGMEDNPTNPGDPGAARIRVSDMAELVDPDPGHEFEQVAEQIYGSMERVSLTTPTMDGFVAQAESVMPGLSKRVMSAFRPEVVPVTTALAMNFAIFDRWFSSVPSSTQPNRFFVHSTTSNGLLSNDKGTLLKGMPQRTIYEDVYEAGLSFGVYYQQIPAMLFFRNMRALKYVQNFNPYDRFKTDAMSGRLPNLVVIEQRYYDVADTPANDDHPTHDISQGQKLIKEVYEILRAGPQWNETLFLITYDEHGGFYDHVPPPAVGVPSPDGVRGPAPHYFNFDRLGVRVPTIAISPWIEKGKVEHRAQGPMPSSEYEHSSIAATIRKLFNLPQPPLTAREAWAGNFAHLISRTTPRTDTPVELPSPPWSLRHSPTVESRPLSEFQEELVVLAKSLRRKLGDWATSEASSKAEEEANPSGMSVGEANFYVRDAVGSFMRRARSQLTAGRHPDSEAAP

>PpNPC1d

MAFRARKFFLPFLLLHLLVWPVRPVHPAHTISGPVKTIVVLVMENRSFDHMLGYLNRRNPNIDGLSGAEFNVLNDGQTGRRTLYVSDTAEFVDPDPGHSYQAIEEQVFGPERKFADPPPMSGFATNAESKLPGMSKNVMRAFRPEVVPVTTALAMEFAVFDRWFASVPSSTQPNRLFVHSATSHGLISNNEDILSAGLPQRTIMEDIADAGLTFGVYYQNLPTLLFYNNMRLAKFSANFIDYEPHFKADAAAGKLPNYVVIEQRYFDVGNSPANDDHPSHDVSQGQMLLKEVYETLRASPQWEEMLLVITYDEHGGFYDHVPTPVTGVPSPDGIEGVAGIYNFTFDRLGVRVPTIAISPWIQKGFVEHKAKGPTAPFSQYEHSSIPATVRKLFNLPSSHLTAREAWAGTFEHLVTQQTMPRKDTPATLASPPYSLRHTAVNEAAPLTEFQSELVLLAASLNGGQMMKAAGERMTVAEANFFVKSSVARFLNAGRAHLRAGGDPNAVINVQGLEYELMHG

>PpNPC1e

MAKIKWCECWRPAKTPNSSPTFATPIKTVVILCMENRSFDHMLGFMKRIQPKIDGLTGTETNPLSLTDANAGVVQVSDQAPYIDPADPGHSFQDITLQIFGSQTETTANPPPMNGFAAQAETEKEGLSKTVMGGFSPDDVPIFKTLVQEFAVVDRWFASVPSSTQPNRQYLHSATSHGLMSNNQQLLLAGLPQKTIFESVEECGLSFGIYYQQVPNTLFYRNLRKLKYVDNFRPYDLTFRDHAKKGTLPNYTVIEPRYFDLPGFPANDDHPVHDVAQGQALIKEVYEALRGSPQWNEILFLITYDEHGGFYDHVPTPIGVPSPDGLVGSASPYSFDFTRLGVRVPTLLISPWIQAGAVLHKPKGPKPTSEFEHSSIPATVKKLFNLPGSFLTKRDEWAGTFETVLTRKTPRTDCPVKLPAPPTTLRTRSAPMNNISALSEFQEELVWLSCSITGKAEVPEYTTIADAAEFVTSAVATFVDAAAAAKAAGVDPDRIFKP

>SbNPC1

MVAAARRDPGTRLLVALLLLALVVSGHCLDAHHRGLKRRRRKHEIHSPVKTVVVVVMENRSFDHILGWLSRTRPDIDGLTGRESNRLNASDPSSPEIFVTDEAGYVDSDPGHGFEDIREQIFGSADTSAVPPPMSGFAQNARGMGLGMPQNVMSGFKPDAVPVYASLADEFAVFDRWFASVPTSTQPNRLFVHSATSHGLTFNARKDLIHGFPQKTIFDSLEENGLSFGIYYQNIPATLFYQSLRRLKHLVKFHQYSLKFKLHAKLGKLPNYVVIEQRYFDCEMFPANDDHPSHDVARGQRFVKEVYETLRASPQWNETALIITYDEHGGFYDHVPTPVVGVPQPDGIVGPDPYYFKFERLGVRVPTFLISPWIEKGTVIHAPNGPQETSQYEHSSIPATVKKLFNLHSNFLTKRDAWAGTFENYLKIRKTPRTDCPEKLPEVTKSLRPFGPKEDSSLSEFQVELIQLASQLNGDHVLNTYPDIGRTMTVGKANRYAEDAVARFLEAGRIALRAGANESALVTMRPALTSRASLSSGLSSEL

>SbNPC2

MAAARARPPPVAALLLLVLLLTGAAGSSTTTSTTSPIKTVVVLVMENRSFDHMLGWMKRLNPEIDGVTGREWNPANTSDPSSGRVYFGDGAAYVDPDPGHSFQEIRQQIFGSDDASGPARMDGFVQQAASIGGGNMTDAVMHGFAPDSVAVYRELVSQFAVCDRWFASVPSSTQPNRLFVHSGTSGGATSNNPTLLAEGYPQRTIFDNLHDAGLSFGVYFQDVPAVLFYRNLRKLKYLLDFHPLRPSFADHARRGVLPNYAVIEQHYLDSKLDPANDDHPSHDVYQGQMLVKYVYETLRASPQWNQTLLVITYDEHGGFFDHVPTPVAGVPSPDGIVGPPPYNFTFDRLGVRVPAILVSPWIDKGTVVHAPTGPTPTSQYEHSSIPATVKKIFNLPQGFLTKRDAWAGTFEGVVQKRTEPRTDCPEQLPTPTRIRQTEADEEAKLSEFQQEIIQLASVLNGDHQLASLQDRIRDEMNVREGIDYMKAAVKRYFEAGASARRMGVDGEQIVKMRPSLTTRIQRP

>SbNPC3a

MAEAPAAATGNSSNNHKIKTVVVVVQENRSFDHMLGWMKTLNPDIDGVTGVETNHVDASDPTSRAVRFSDGAEYVDPDPGHSMQAIYEQVYGTPFVDAATTPITPPGVPAAPPMSGFAQQAEKEKPGMSGTVMSGFRPDAVPVYRELVKEFAVCDRWFASNPASTQPNRLFVHSATSHGLVSNDTKALVAGLPQRTIFDALHDEGFSFGIYYQYPPSTLFYRNLRQLKYVGSFHAFDLDFRRHCREGKLPNYVVVEQRYFDLEILPGNDDHPSHDVAEGQRFIKEVYEALRSGPQWEETLLVVTYDEHGGFYDHVPTPAGAGVVPSPDGIVSASPFFFGFDRLGVRVPALLVSPWIEPGTVLHGPSGPYPTSEFEHSSIPATVKKLFNLRSFLTKRDAWAGTFDCVLTRDTPRTDCPRTLPEPVKLRRTVAAEHAPLSEFQEELVQLAAVLNGDHTKDSYPHKLVEGMTVAEAARYCVDAFKAFRDECEKCKKRGEDGSHIPTVKPSASGKDKDKSKSKSKSSFVSKALLACLPCARPSS

>SbNPC3b

MADKIKTVVVLVQENRSFDHMLGWMKSLNSEIDGVTGAEVNYTVAGDATSTAVHFGNASQYVDPDPGHSFMAIYEQIYGDAFTWGVTAPATKPGVTVPPMSGFAQQAEAEKPGTPHAVMNGFRPDAVPVYRELVGEFAVCDRWFASVPTSTQPNRMFVHSATSHGLVGNDKKLLREGMPQRTIFDALHDAGHSFGIYYQFPPAVLLYRNMRQLKYIGKFHEYELHFKRHCREGKLPNYVVIEQRYLDWKLLPGNDDHPSHDVAHGQRLVKEVYEALRSSPQWNEILFVITYDEHGGFFDHVPTPVDGVPSPDGIVSAAPINFAFDRLGVRVPAMFISPWIEPGTVIHRPPSGPEPTSQYEHSSIPATVKKIFGLKEFLTKRDAWAGTFEHVLTRATPRTDCPETLPEPVRLREAKAEEDQRREVSEFQAELVQLGAALNGDHATEAYESDKLVKGMTVAEASDYCRAAFARFREECQRCHEGGMDECHVPALPPPTASKLCGCLPCFSAS

>SbNPC6

MHPWPTIQRTVTRRMPPKHASPCPPSVPAPSSSTCTSTSTAGISSNTPVPDMATRSTHRRPLLLILLFLLLFAAVNGSARPSTSPIKNVVVLALENRSFDHMLGWMRRLLGLPVDGLTGAECNPNSTNSTTSSICVSADADLVVPDDPGHSFEDVLEQVFGNGNISAAQPSMSGFVRSALSVNALLSSAVMRAFRPSLLPTFSALAPAFAVFDRWFSSIPGPTQPNRLFLYSATSRGAVAHDKLDLLLGYPQRTIFESLAADGHDFAVYFKTIPTVLFYRRLRALRYAARSFHRYDAAFKDHARRGVLPALSVIEPRYFDLTGTPADDDHPAHDVANGQRLVKDVYEALRASPQWNQTLLIVTYDEHGGFYDHVSTPTAGVPSPDGIRGPPPFFFKFDRLGVRVPTIMVSPWIKKGTVVGRAVGPTDTSEFEHSSIPATIKKIFNLSSDFLTKRDAWAGTFEHIFTELDQPRTDCPETLPEVPFVRPTPPKEHGWLSDFQRELVELASFLNGDYMLTSLAQESRKKKMTVKQADAYVRRAITSFLQASKQAVRLGANESAIVTMRSSLTSKSSSSSSP

>SmNPC1a

MKWCLVPLFLLLLSPGTNAAQPSGRIKTIVVLVQENRSFDHMLGWLKKLNPEIDGLTGKESNPMNLTDPSSGTVFVSDKAEFVDPDPGHSFGAIRDQVFGFGSTSQNPAPMNGFAQQAEIIQKNLSQRVMSSFRPELVPAYTALAMEFAICDKWFASVPASTQPNRLYIHSATSHGAVSNVRSDLVKGFPQKTIFESIDQDKLSFGIYYQNIPATNLRSPKYLGKFHNYGLFKTHAKQGKLPNYVVVEQRYYDTKATPANDDHPSHDVAEGQKFIKEVYETLRSSPQWNETLLVITYDEHGGFFDHVSTPMDNVPNPDGLRGGDDDHFNFDRLGVRVPAIFVSPWIDKGTVIHRPNGPTKDSQYEHSSIPATVKKIFNLTQPFLTKRDAWAGTFETVLSSTRTTPRTDCPVTLPSSPWSLRHSPPNEEGRLTEFQVEMVGLASQLNGDYGKSGYPNLGASMTVKYASDYVDRAVEGIMRAGKVALQSGEDPNALIEVLPTSESRKTGT

>SmNPC1b

MVLWLLLSWNLCLAHQQQWQQQLGWRHKKKISGPIKTVVVLVMENRSFDHMLGWLKKLNPEIDGLQGNESNPLSTTDPASRKIFVADTAEFVDPDPGHSFQAITEQVFGSNDTSAIPPPMNGFAQQAESMVEGFSETVMKGFRPELVPVYTALAMEFAVFDRWFASTPTSTQPNRLYVHSATSYGEISNVKKDLVKGFPQKTIYDSLDEDGLSFGIYYQNIPACLFFKNLRKLKYLTKYHSYRAAFKLHARLGMLPNVAVIEQRYFDLDLTPANDDHPSHDVSEGQKLVKEVYEALRSSPQWNEVLFVITYDEHGGFYDHVPTPNVGVPNPDGVLGPEPGFFDFDRLGVRVPTIMVSPWIEKGTVVHEPNGPTPTSQFEHSSLAATIKKLFDLKSDFLTKRDAWAGTFESVVSGRSSPRTDCPETLPTPPWSLRHRAVDEDAKLTEFQEELVQLASQLNGDHRLNGYPNFGKGMSVRQASDYVESAVRKFLDEGRLALKSGGQADDTTVLDVNIRKSTV

>GrNPC1

MENRSFDHLLGWLKSTRPDIDGLSGTESNPVNVADPNSPFISVSDDALFVDSDPGHSFQAIREQIFGSNDSSADSAPMNGFAQQAESMGEGMGRTVMSGFKPSRLPVYTKLANEFGVFDRWFASVPASTQPNRFYVHSATSFGATSNVKKDLIHGFPQKTIFDSLDENGLSFGIYYQNIPATLFFKSLRKLKFLTKFHNYALKFRLHARLGKLPNYVVVEQRYFDVKEFPANDDHPSHDVARGQRFVKEVYEILRSSPQWKEMALLITYDEHGGFYDHVPTPVSGVPNPDGIVGPDPFYFKFNRLGVRVPTLLVSPWIDKATVIHEPTGPTPSSQFEHSSIPATVKKLFNLNSNFLTKRDAWAATFENYFKLRTTPRTDCPETLPEVTTSLRPWGPKEDASLSEFQVELVQLASQLNGDYVLNTYPYIGKSMRVGEANRYVEDAVKRFLEAGKAAIRAGANESAIVTMRPSLTSRIEDRGQHVEAY

>GrNPC2

MFKPANTAIFFFFVLFNSFSCHGGPIKTIVVLVMENRSFDHMLGWMKKINPQINGVDGTEWNPLSTTDPNSKKLFFQNQAQFVDPDPGHSFQAIREQIFGSNDTSANPPPMNGFAQQAYSMDPSTTMSQNVMNGFDPEMVAVYKSLVSEFAVFDRWFASVPSSTQPNRLYVHSATSAGATSNIPALLVKGYPQRTIFENLDDAGISWGIYYQNIPATLFYKNLRKLKYLFRFRPYGVTFKKHAQEGKLPGYVVVEQRYMDTKLEPANDDHPSHDVYQGQMFVKEVYETLRASPQWNQTLLIITYDEHGGFYDHVATPVTGVPSPDGIVGPEPFFFHFDRLGVRVPTIMVSPWIDKGTVVHGANGRPFPTSEFEHSSIPATVKLLFNLTSPFLTKRDEWAATFESILRTRSDPRTDCPETLPTPARIRRGEAIEEAKLSEFQQELVQLAAVLKGDHILTSYPERIGKDMSVKEGKEYMEDAVKRFFEAGHYAKKMGVDGEHIVQMKPSLTTRSSKPSSQHP

>GrNPC3a

MAVETSSATPSPIKTVVVLVQENRSFDHMLGWFKTINPEIGGVTGSESNPISTSDPNSTQITFKDTAGYVDPDPDHSFQAIYEQVSGKTWDTNNPDPNPEIKMNGFVQNAERTTPGLSETVMNGFKPEAVPVFKQLVTEFAVCDRWFASLPASTQPNRLYVHSATSHGAMSNNTQQLIEGFPQKTIFESLEENGYSFGIYYQSFPSTLFYRKLRHLKYVDNFHQYDLSFKRHCKDGKLPNYVVIEPRYFDILTAAANDDHPSHDVSEGQKLVKEIYEALRSSPQWNEILFLVIYDEHGGFYDHVPTPTGVPSPDDIVGPEPYNFKFDRLGCRVPAIMVSPWIEPETVLHRPSGPDPTSEFEHSSIAATLKKIFNLKEFLTKRDAWAGSFDIVVNRSTPRTDCPEKLAEPVKMRDTDAKETAKLSDFQEELVQAAAALKGDPFNLVENMTVSSGLKYVEDAFKKFYDDGQKAKEINEVEDTVSADASTRRTTASKTFMQKVFSCLVCDR

>GrNPC3b

MAVETSSATPSPIKTVVVLVQENRSFDHMLGWFKTINPEIGGVTGSESNPISTSDPNSTQITFKDTAGYVDPDPDHSFQAIYEQVSGKTWDTNNPDPNPEIKMNGFVQNAERTTPGLSETVMNGFKPEAVPVFKQLVTEFAVCDRWFASLPASTQPNRLYVHSATSHGAMSNNTQQLIEGFPQKTIFESLEENGYSFGIYYQSFPSTLFYRHLKYVDNFHQYDLSFKRHCKDGKLPNYVVIEPRYFDILTAAANDDHPSHDVSEGQKLVKEIYEALRSSPQWNEILFLVIYDEHGGFYDHVPTPTGVPSPDDIVGPEPYNFKFDRLGCRVPAIMVSPWIEPETVLHRPSGPDPTSEFEHSSIAATLKKIFNLKEFLTKRDAWAGSFDIVVNRSTPRTDCPEKLAEPVKMRDTDAKETAKLSDFQEELVQAAAALKGDPFNLVENMTVSSGLKYVEDAFKKFYDDGQKAKEINEVEDTVSADASTRRTTASKTFMQKVFSCLVCDR

>GrNPC3c

MAVETSSATPSPIKTVVVLVQENRSFDHMLGWFKTINPEIGGVTGSESNPISTSDPNSTQITFKDTAGYVDPDPDHSFQAIYEQVSGKTWDTNNPDPNPEIKMNGFVQNAERTTPGLSETVMNGFKPEAVPVFKQLVTEFAVCDRWFASLPASTQPNRLYVHSATSHGAMSNNTQQLIEGFPQKTIFESLEENGYSFGIYYQSFPSTLFYRKLRHLKYVDNFHQYDLSFKRHCKDGKLPNYVVIEPRYFDILTAAANDDHPSHDVSEGQKLVKEIYEALRSSPQWNEILFLVIYDEHGGFYDHVPTPTGVPSPDDIVGPEPYNFKFDRLGCRVPAIMVSPWIEPETVLHRPSGPDPTSEFEHSSIAATLKKIFNLKEFLTKRDAWAGSFDIVVNRSTPRTDCPGKFNYLNTLQLFFFRSKVIESYNNNYCIKM

>GrNPC4a

MYVYISQPIPNQQRTYKNRTMVSQGSNSASSYPIKTIVILVQENRSFDHMLGWFKSLNPEIDGVTGSESNPISTSDPNSPMVFFKDNSEYVDPDPAHSIQAIYEQVFGHPWSSDLPNPPHEPTMNGFAQNAERTEKGMAEAVMKGFKPDAVPVYKELASKFGICDRWFASVPASTQPNRMFVHSATSYGQTSNDAIKLIKGFPQKTIFESLDESGFSFGIYYQYPPSTLFFRNLRQMKYLKNFHQFDLHFKKHCEEGKLPNYVVVEQRYFDLLSVPANDDHPSHDVSEGQKFVKEVYEALRSSPQWKEMLLVITYDEHGGFYDHVPTPTTGVPSPDDIVGPEPYHFKFDRLGVRVPTFFVSPWIEPGTVIHRPLGPYPTSEFEHSSIPATVKKIFNLKEFLTKRDAWAATFEGVINRKNPRVDCPVTLPEPVKMRPTEAKETAKLSDFQKELVQMAAVLNGDHKSDMYPHKLVEKMTVAEAAKYVNGAFNKFCDECQRGGIHESEIVELGKQVERPKGRSFIYKFFKCLVCHD

>GrNPC4b

MYVYISQPIPNQQRTYKNRTMVSQGSNSASSYPIKTIVILVQENRSFDHMLGWFKSLNPEIDGVTGSESNPISTSDPNSPMVFFKDNSEYVDPDPAHSIQAIYEQVFGHPWSSDLPNPPHEPTMNGFAQNAERTEKGMAEAVMKGFKPDAVPVYKELASKFGICDRWFASVPASTQPNRMFVHSATSYGQTSNDAIKLIKGFPQKTIFESLDESGFSFGIYYQYPPSTLFFRNLRQMKYLKNFHQFDLHFKKHCEEGKLPNYVVVEQRYFDLLSVPANDDHPSHDVSEGQKFVKEVYEALRSSPQWKEMLLVITYDEHGGFYDHVPTPTTGVPSPDDIVGPEPYHFKFDRLGVRVPTFFVSPWIEPGTGPYPTSEFEHSSIPATVKKIFNLKEFLTKRDAWAATFEGVINRKNPRVDCPVTLPEPVKMRPTEAKETAKLSDFQKELVQMAAVLNGDHKSDMYPHKLVEKMTVAEAAKYVNGAFNKFCDECQRGGIHESEIVELGKQVERPKGRSFIYKFFKCLVCHD

>GrNPC6a

MGESKTSPPPSFSFIFSLFLTVACLFTPLGAQQQSPIKTIVVLVMENRSFDHMLGWMKQHVNPSINGVTGDECNPVSTKNPNLESICFTDDAEFVDPDPGHSFEAVEQQVFGSSTIPSMSGFVEQALSMSKNLSETVMKGFRPESVPVYAALVKEFAVFDRWFSSIPGPTQPNRLFVYSATSHGSTSHVKKQLAHGYPQKTIFDSLHENGKDFGVYFQNIPTTLFYRSLRKLKYVFKFHQFDLKFKKDARKGKLPSLTVIEPRYFDLKGLPANDDHPSHDVANGQKLVKEVYEILRASPQWNQTLLVITYDEHGGFYDHVHTPYINVPSPDGNTGPAPSFFKFDRLGVRVPTIMVSPWIKKGTGKLNVDAFRCMRLLY

>GrNPC6b

MGESKTSPPPSFSFIFSLFLTVACLFTPLGAQQQSPIKTIVVLVMENRSFDHMLGWMKQHVNPSINGVTGDECNPVSTKNPNLESICFTDDAEFVDPDPGHSFEAVEQQVFGSSTIPSMSGFVEQALSMSKNLSETVMKGFRPESVPVYAALVKEFAVFDRWFSSIPGPTQPNRLFVYSATSHGSTSHVKKQLAHGYPQKTIFDSLHENGKDFGVYFQNIPTTLFYRSLRKLKYVFKFHQFDLKFKKDARKGKLPSLTVIEPRYFDLKGLPANDDHPSHDVANGQKLVKEVYEILRASPQWNQTLLVITYDEHGGFYDHVHTPYINVPSPDGNTGPAPSFFKFDRLGVRVPTIMVSPWIKKGTVISGPKGPFPNSEFEHSSIPATIKKMFNLSSNFLTHRDAWAGTFEHVVGELSSPRTDCPEKLPEAALLRTTEANEDAGLSEFQSEVVQLASVLNGDHFLSSFAEEMRTKMSVKGAHEYVKGAVSRFIRASKEAIKLGANESTIVDMRSSLTTRSSSIHN

>GrNPC6c

MGESKTSPPPSFSFIFSLFLTVACLFTPLGAQQQSPIKTIVVLVMENRSFDHMLGWMKQHVNPSINGVTGDECNPVSTKNPNLESICFTDDAEFVDPDPGHSFEAVEQQVFGSSTIPSMSGFVEQALSMSKNLSETVMKGFRPESVPVYAALVKEFAVFDRWFSSIPGPTQPNRLFVYSATSHGSTSHVKKQLAHGYPQKTIFDSLHENGKDFGVYFQNIPTTLFYRSLRKLKYVFKFHQFDLKFKKDARKGKLPSLTVIEPRYFDLKGLPANDDHPSHDVANGQKLVKEVYEILRASPQWNQTLLVITYDEHGGFYDHVHTPYINVPSPDGNTGPAPSFFKFDRLGVRVPTIMVSPWIKKGTVISGPKGPFPNSEFEHSSIPATIKKMFNLSSNFLTHRDAWAGTFEHVVGELSSPRTDCPGIFFFNFMLQGLLNE

>GrNPC6d

MERSFSFIFLLFILPFVVSQESPIKTIVVLVMENRSFDHMVGWMKQGINPTINGVTGNECNPISTKTPNPKSVCFTNDAQFVDPDPGHSFEAVEQQVFGSTPSSFPSMSGFVEQAFSISPNMSETVMKGFKPEAVPIYASLVKEFAVFDRWFSSIPGPTQPNRLFVYSATSHGSTSHVKKQLAQGYPQKTIFDSLHENGKDFGVYFQNIPTTLFYRNLRKLKYVFKFHQFDLKFKKDALNGKLPSLSVIEPRYFDLKGLPANDDHPSHDVANGQKLVKEVYETLRASPQWNETLLVITYDEHGGFYDHVKTPFVNVPNPDGNTGPAPSFFKFDRLGVRVPTIMVSPWIKKGTVISGPKGPTPNSEFEHSSIPATIKKIFNLSSNFLTHRDAWAGTFEDVVSHLTSPRTDCPETLSDVVPLRTTEAKEDAALSEFQSEVVQLAAVLNGDHFLSSFPDEMSKKMTVKEAHEYTKGAVSRFIRASKEALKLGAAESAIVDMRSSLTTRSSNP

>GaNPC1a

MSSGRRVPFTLVLFFCLFVSSHSLTSNYNFRKKHKIKGPIKTLVVLVMENRSFDHLLGWLKSTRPDIDGLSGTESNPVNVTDPNSPFISVSDDALFVDSDPSHFFQAIREQIFGSNNSSADSAPMNGFAQQAESMGEGMGRTVMSRFKPSRLPVYTKLANEFGVFDRWFASVPASTLPNSFGIYYQNIPTTLFFKSLRKLKFLTKFHNYALKFRLHARLGKLPNYVVVEQRYFDVKEFPANDDHPSHEWRVGRGRGFYDHVPTPVSGVPNPEGIVGPDPFYFKFNRLGVRVPTLLVSPWIDKATETLPEVTTSLRPWGPKEDTSLSEFQVELVQLASQLNGDYVLNTYPYTGKSMRVGEANRYVEDAVKRFLEAGKAAIRADANESEIVTMRPSLTSRIEDQSQHVEAY

>GaNPC1b

MSSGRRVPFTLVLFFFLLVSSHSLASNYNFRKKHKIKGPVKTLVVLVMENRSFDHLLGWLKSTRPDIDGLSGTESNPVNVADPNSPFISVSDDALFVDSDPGHSFQAIREQIFGSNDSSADSAPMNGFAQQAESMGEGMGRTVMSGFKPSRLPVYTKLANEFGVFDRWFASVPASTQPNRFYVHSATSFGATSNVKKDLIHGFPQKTIFDSLDENGLSFGIYYQNIPATLFFKSLRKLKFLTKFHNYALKFRLHARLGKLPNYVVVEQRYFDVKEFPANDDHPSHDVARGQRFVKEVYEILRSSPQWKEMALLITYDEHGGFYDHVPTPVSGVPNPDGIIGPDPFYFKFNRLGVRVPTLFVSPWIDKATVIHEPTGPTPSSQFEHSSIPATVKKLFNLNSNFLTKRDAWAATFENYFKLRTTPRTDCPETLPEVTTSLRPWGPQEDASLSEFQVELVQLASQLNGDYVLNTYPSIGKSMRVGEANRYVDDAVKRFLEAGKAAIRAGANESAIVTMRPSLTSRIEDRSQHVEAY

>GaNPC2

MENRSFDHMLGWMKKINPEINGVDGTEWNPLSTTDPNSKKLFFQNQAQFVDPDPGPSFQAIREQIFGSNDTSTNPPPMNGFAQQAYSMDPSTTMSQNVMNGFDPEMVPVYKSLVSEFAVFDRWFASVPSSTQPNRLYVHSATSAGATSNIPALLVKGYPQRTIFENLDAAGISWGIYYQNIPATLFYKNLRKLKYLFRFRPYGVTFKKHAQEGKLPGYVVVEQRYMDTKLEPANDDHPSHDVYQGQMFVKEVYETLRASPQWNQTLLIITYDEHGGFYDHVATPVTGVPSPDGIVGPEPFFFHFDRLGVRVPTIMVSSWIDKGTVVHGANGRPFPTSEFEHSSIPATVKLLFNLTSPFLTKRDEWAATFESILRTRSDPRTDCPETLPTPARIRRGEANEEAKLSEFQQELVQLAAVLKGDYILTSYPERIGKEMSVKEGKEYMEDAVKRFFEAGHFAKKMGVDGEHIVQMKPSLTTRSTKPSSQHP

>GaNPC3

MAVETSSATPSPIKTVVVLVQENRSFDHMLGWFKTINPEIDGVTGSESNPISTSDPNSTQITFKDTAAYVDPDPDHSFQAIYEQVSGKTWDTSNPDPNPGIKMNGFVQNAERTTPGLSETVMNGFKPEAVPVFKQLVTEFAVCDRWFASLPASTQPNRLYVHSATSHGAMSNNTQQLIEGFPQKTIFESLEENGYSFGIYYQSFPSTLFYRKLRHLKYVDNFHQYDLSFKRHCKDGKLPNYVVIEPRYFDILTAAANDDHPSHDVSEGQKLVKEIYEALRSSPQWNEILFLVIYDEHGGFYDHVPTPTGVPSPDDIVGPEPYNFKFDRLGCRVPAIMVSPWIEPGTVLHRPSGPDPTSEFEHSSIAATLKKIFNLKEFLTKRDAWAGSFDIVVNRSTPRTDCPEKLAEPVKMRDSDAKETAKLSDFQEELVQAAAALKGDPFNLVENMTVSSGLKYVEDAFKKFYDDGQKAKEINEVEDTVSADASTRRTTASKTFMQKVFSCLVCDR

>GaNPC4

MVSQGSNSASSYPIKTIVILVQENRSFDHMLGWFKSLNPEIDGVTGSESNPISTSDPNSPMVFFKDNSEYVDPDPAHSIQAIYEQVFGQPWSSDLPNPPHEPTMNGFAQNAERTEKGMAEAVMKGFKPDAVPVYKELASKFGICDRWFASVPSSTQPNRMFVHSATSYGQTSNDAIKLIKGFPQKTIFESLDESGFSFGIYYQYPPSTLFFRNLRQMKYLKNFHQFDLHFKKHCEEGKLPNYVVVEQRYFDLLSVPANDDHPSHDVSEGQKFVKEVYEALRSSPQWREMLLVITYDEHGGFYDHVPTPTTGVPSPDDIVGPEPYHFRFDRLGVRVPTFFVSPWIEPGTVIHRPSGPYPTSEFEHSSIPATVKKIFNLKEFLTKRDAWAATFEGVINRKNPRVDCPVTFPEPVNMRPTEAKETVKLSDFQKELVQMAAVLNGDHKSGMYPHKLVEKMTVAEAAKYVNGAFDKFCDECQRGGIHESEMVELGKQVERPKGRSFIDKFFKCLVCHD

>GaNPC6a

MGESKASPPPSFSFIFSLFLTVACLFTPLGAQQQSPIKTIVVLVMENRSFDHMLGWMKQHVNPSINGVTGDECNPVSTKNPNPESICFTDDAEFVDPDPGHSFEAVEQQVFGSSTIPSMSGFVEQALSMSKNLSETVMKGFRPESVPVYAALVKEFAVFDRWFSSIPGPTQPNRLFVYSATSHGSTSHVKKQLAHGYPQKTIFDSLHENDKDFGVYFQNIPTTLFYRNLRKLKYVFKFHQFDLKFKKDARKGKLPSLTVIEPRYFDLKGLPANDDHPSHDVANGQKLVKEVYEILRASPQWNQTLLVITYDEHGGFYDHVHTPYINVPSPDGNTGPAPSFFKFDRLGVRVPTIMVSPWIKKGTVISGPKGPFPNSEFEHSSIPATVKKMFNLSSNFLTHRDAWAGTFERVVGELSSPRTDCPEKLPEAAPLRTTAANEDAGLSEFQSEVVQLASVLNGDHFLSGFAEEMHTKMSVKGAHEYVKGAVSRFIRASKEAIKLGADESAIVDMRSSLTTRSSSIHN

>GaNPC6b

MEGSFSFIFLLFLLPFVVSQESPIKTIVVLVMENRSFDHMVGWMKQSINPTINGVTGNECNPISTKTPNPKSICFTNDAQFVDPDPGHSFEAVEQQVFGSTLSSFPSMSGFVEQAFSISPNMSETVMKGFKPEAVPIYATLVKEFAVFDRWFSSIPGPTQPNRLFVYSATSHGSTSHVKKQLAQGYPQKTIFDSLHENGKDFGVYFQNIPTTLFYRNLRKLKYVFKFHQFDLKFKKDALNGKLPSLSVIEPRYFDLKGLPANDDHPSHDVANGQKLVKEVYETLRASPQWNETLLVITYDEHGGFYDHVKTPFVNVPNPDGNTGPAPSFFKFDRLGVRVPTIMVSPWIKKGTVISGPKGPTPNSEFEHSSIPATIKKIFNLSSNFLTHRDAWAGTFEDVVSHLTSPRTDCPETLPDVVPLRATEAKEDAALSEFQSEVVQLAAVLNGDHFLSSFPDEMSKKMTVKEAHEYTKGAISRFIRASKEALKLGAAESAIVDMRSSLTTRSSNP
