# Supplementary material for: Structural, expression and evolutionary analysis of the non-specific phospholipase C gene family in Gossypium hirsutum
Source: BMC Genomics. 2017 Dec 19;18:979. doi: 10.1186/s12864-017-4370-6 (PMC5738194; doi:10.1186/s12864-017-4370-6)
Supplement: Supplementary file 3 — List of primers used in quantitative real time-PCR expression analysis (DOC 39 kb) [file 12864_2017_4370_MOESM3_ESM.doc]

**Additional File3: Table S2** List of primers used in quantitative real time-PCR expression analysis.

| Primer name | Sequence(5'–3') |
| --- | --- |
| GhNPC1a-F: | TCCCCACTCTCTTGGTCTCTCC |
| GhNPC1a-R: | TAAGGGTAAGTATTGAGGACG |
| GhNPC1b-F: | TCCCCACTCTCTTGGTCTCTCG |
| GhNPC1b-R: | GAAGGGTAAGTATTGAGGACA |
| GhNPC2a-F: | TGTTCTCTTCAACAGCTTCAGCT |
| GhNPC2a-R: | ATTCTGAGACATAGTGGTAGATA |
| GhNPC2b-F: | CTTCTTCTTCTTCAGCTGCCATG |
| GhNPC2b-R: | GTTCTGAGACATAGTGGTAGATG |
| GhNPC3a-F: | GCAGTTGAAACAAGCTCTGCAACT |
| GhNPC3a-R: | GAGTTGTACGTTCAGCATTTTGTAC |
| GhNPC3b-F: | TATCAGGAAGCTTAGGCACTTGA |
| GhNPC3b-R: | AGGCTCAGGACCGACAATATCATC |
| GhNPC4-R: | GTATATATATCTCAACCCATTCC |
| GhNPC4-F: | TTAGGGAGGTCAGAGCTCCAA |
| GhNPC6a-F: | GAACTCTGAATTCGAGCACTCA |
| GhNPC6a-R: | TCAACAATGGCAGATTCATC |
| GhNPC6b-F: | GAACTCCGAATTCGAGCACTTG |
| GhNPC6b-R: | TCAACAATGGTGGATTCATT |
| GhNPC6c-F: | AGCATAAACCCAACCATCAAC |
| GhNPC6c-R: | TTGCATAAATAGGGACAGCTTCTGGTT |
| GhNPC6d-F: | GGCATAAACCCAACCATCAAT |
| GhNPC6d-R: | ATGCATAAATAGGGACAGCTTCTGGTC |
