# Supplementary material for: Structural, expression and evolutionary analysis of the non-specific phospholipase C gene family in Gossypium hirsutum
Source: BMC Genomics. 2017 Dec 19;18:979. doi: 10.1186/s12864-017-4370-6 (PMC5738194; doi:10.1186/s12864-017-4370-6)
Supplement: Supplementary file 4 — The CDS sequences of GhNPCs (DOC 39 kb) [file 12864_2017_4370_MOESM4_ESM.doc]

**Additional File4: Data Set S2.** The CDS sequences of *GhNPC*s.

>GhNPC1a

ATGGAAAACCGTTCTTTTGACCATCTTTTGGGTTGGCTCAAATCGACCCGACCCGACATAGACGGTCTCTCCGGCACAGAGTCAAACCCAGTCAACGTCGCCGACCCCAACTCCCCCTTTATCTCTGTCTCCGACGACGCTCTCTTTGTCGACTCCGACCCAGGCCACTCTTTTCAAGCGATCAGAGAACAGATTTTCGGGTCGAACGACAGCTCTGCTGACTCGGCTCCAATGAACGGCTTCGCCCAACAAGCGGAGAGCATGGGTGAAGGAATGGGAAGAACCGTGATGAGCGGATTTAAACCGAGTCGGTTACCGGTTTACACGAAGTTAGCGAACGAGTTCGGCGTTTTCGACCGGTGGTTTGCTTCGGTTCCGGCTTCGACTCAACCGAACAGGTTTTACGTTCATTCAGCAACGTCGTTTGGGGCGACGAGTAATGTCAAGAAAGACCTCATCCATGGATTCCCCCAAAAGACGATTTTCGATTCGTTGGACGAAAATGGCCTCAGCTTCGGCATTTATTACCAAAACATCCCGGCCACCCTTTTCTTCAAAAGCCTAAGGAAATTAAAGTTCTTGACCAAATTCCACAACTACGCTTTGAAGTTCCGGCTTCACGCGCGGCTTGGGAAGCTGCCGAATTACGTGGTGGTGGAGCAGCGTTACTTCGACGTGAAGGAGTTTCCGGCGAACGACGACCACCCGTCGCATGACGTGGCGCGTGGGCAGAGGTTCGTGAAGGAGGTGTACGAGATACTGAGAAGTAGCCCGCAGTGGAAAGAGATGGCGCTTCTGATCACGTACGATGAGCACGGAGGGTTTTATGATCACGTGCCGACACCTGTGTCGGGTGTTCCTAACCCGGACGGAATAGTTGGACCCGACCCGTTTTATTTCAAGTTCAATAGGCTTGGTGTTAGGGTCCCCACTCTCTTGGTCTCTCCCTGGATCGATAAGGCAACTGTGATCCACGAGCCAACTGGGCCAACACCGTCTTCCCAATTTGAACATTCTTCCATCCCTGCAACTGTGAAGAAGCTCTTCAACCTGAATTCAAATTTCCTGACAAAGAGGGATGCCTGGGCTGCTACATTTGAAAATTATTTTAAGCTACGTACTACTCCACGAACTGACTGTCCTGAAACTCTTCCAGAGGTGACGACTTCATTGAGGCCATGGGGGCCAAAAGAAGATGCTAGCCTCTCAGAATTCCAAGTTGAGCTGGTTCAGCTTGCATCACAGCTCAATGGTGATTACGTCCTCAATACTTACCCTTATATTGGGAAAAGCATGCGAGTAGGTGAAGCCAACCGATACGTAGAGGATGCAGTCAAGAGGTTCCTGGAAGCCGGAAAGGCTGCTATAAGAGCCGGAGCTAATGAATCTGCAATTGTTACGATGAGGCCTTCTCTTACCAGTCGAATCGAGGATCGGGGTCAACATGTGGAAGCCTATTAG

>GhNPC1b

ATGGAAAACCGTTCTTTTGACCATCTTTTGGGTTGGCTCAAATCGACCCGACCCGACATAGACGGTCTCTCCGGCACAGAGTCAAACCCAGTCAACGTCGCCGACCCCAACTCCCCCTTTATCTCTGTCTCCGACGATGCTCTCTTTGTCGACTCCGACCCAGGCCACTCTTTTCAAGCGATCAGAGAACAGATTTTCGGGTCGAACGACAGCTCTGCTGACTCGGCTCCTATGAACGGCTTCGCCCAACAAGCGGAGAGCATGGGTGAAGGAATGGGTAGAACCGTGATGAGCGGATTTAAACCGAGTCGGTTACCGGTTTACACGAAGTTAGCGAACGAGTTCGGCGTTCTCGACCGGTGGTTTGCTTCGGTTCCGGCTTCGACTCAACCTAACAGGTTTTACGTTCATTCAGCAACGTCGTTTGGGGCGACGAGTAATGTCAAGAAAGACCTCATCCATGGATTCCCCCAAAAGACGATTTTCGATTCGTTGGACGAAAACGGCCTCAGCTTCGGCATTTATTACCAAAACATCCCTGCCACCCTTTTCTTCAAAAGCCTAAGGAAATTAAAGTTCTTGACCAAATTCCACAACTACGCTTTGAAGTTCCGGCTCCACGCGCGGCTTGGGAAGCTGCCGAATTACGTGGTGGTGGAGCAGCGTTACTTCGACGTGAAGGAGTTTCCGGCGAACGACGACCACCCGTCGCATGACGTGGCGCGTGGGCAGAGGTTCGTGAAGGAGGTGTACGAGATACTGAGAAGTAGCCCGCAGTGGAAAGAGATGGCGCTTCTGATCACGTACGATGAGCACGGAGGGTTTTATGATCACGTTCCGACACCTGTCTCGGGTGTTCCTAACCCGGACGGAATAATTGGACCCGACCCGTTTTATTTCAAGTTCAATAGGCTTGGTGTTAGGGTCCCCACTCTCTTGGTCTCTCGCTGGATCGATAAGGCAACTGTGATCCACGAGCCAACTGGGCCAACACCGTCTTCCCAATTTGAACATTCTTCCATCCCTGCAACTGTGAAGAAGCTCTTCAACCTGAATTCAAATTTCCTGACAAAGAGGGATGCCTGGGCTGCTACATTTGAAAATTATTTTAAGCTGCGTACTACTCCACGAACTGACTGTCCTGAAACTCTTCCAGAGGTGACGACTTCATTGAGGCCATGGGGGCCACAAGAAGATGCTAGCCTCTCAGAATTCCAAGTTGAGCTGGTTCAGCTTGCATCACAGCTCAATGGTGATTATGTCCTCAATACTTACCCTTCTATTGGGAAAAGCATGCGAGTAGGTGAAGCCAACCGATACGTAGAAGATGCAGTCAAGAGGTTCCTGGAAGCCGGAAAGGCTGCTATAAGAGCCGGAGCTAATGAATCTGCAATTGTTACAATGAGGCCTTCTCTTACCAGTCGAATCGAGGATCGGAGTCAACATGTGGAAGCCTATTAG

>GhNPC2a

ATGTTCAAACCTGCTAACACTGCCATCTTCTTCTTCTTTGTTCTCTTCAACAGCTTCAGCTGCCATGGCGGTCCAGTCAAAACTATTGTGGTCCTAGTGATGGAGAATCGCTCGTTCGATCACATGCTAGGATGGATGAAGAAAATCAACCCTCAAATCAACGGTGTTGATGGAACTGAATGGAACCCTTTGTCCACCACTGATCCTAACTCCAAAAAGTTGTTCTTCCAGAACCAAGCTCAGTTCGTGGATCCAGATCCTGGTCACTCTTTCCAAGCCATAAGGGAGCAGATTTTCGGCTCTAATGACACCTCCGCCAATCCTCCCCCCATGAATGGCTTCGCCCAACAAGCTTACTCCATGGACCTATCTACCACTATGTCTCAGAATGTCATGAATGGATTTGACCCTGAAATGGTTGCCGTTTACAAGTCTCTTGTCTCAGAATTTGCTGTCTTTGATCGGTGGTTCGCCTCCGTACCGTCATCCACACAACCGAATCGTCTGTACGTCCACTCGGCGACGTCGGCGGGGGCGACGAGCAACATTCCGGCACTCCTGGTAAAGGGTTATCCCCAAAGGACGATCTTCGAGAACCTGGACGACGCCGGAATATCCTGGGGAATATACTACCAGAACATCCCAGCTACATTGTTCTACAAAAACCTCAGGAAACTCAAATACTTATTTAGATTCCGTCCGTACGGTGTGACATTCAAAAAACATGCGCAAGAAGGGAAGCTGCCGGGCTACGTTGTGGTGGAGCAGCGGTACATGGACACTAAGCTGGAGCCCGCCAACGATGACCATCCGTCGCACGATGTGTATCAAGGGCAGATGTTTGTGAAGGAGGTGTACGAGACATTGAGAGCTAGCCCGCAGTGGAACCAAACTTTGTTGATCATTACATATGATGAACATGGTGGGTTTTATGATCATGTGGCTACGCCCGTCACTGGAGTGCCTAGCCCTGATGGCATTGTGGGTCCCGAACCATTTTTCTTTCATTTCGACAGATTGGGGGTTAGGGTTCCGACCATCATGGTCTCTCCTTGGATTGATAAGGGCACTGTTGTTCATGGGGCAAATGGAAGGCCATTTCCCACATCGGAATTCGAGCATTCCTCCATTCCGGCAACAGTAAAGTTGTTGTTCAACCTCACCTCTCCATTCCTCACCAAGAGGGACGAATGGGCTGCCACTTTTGAGTCCATTCTCCGAACTCGGTCTGACCCCAGAACTGATTGTCCAGAGACACTGCCAACGCCGGCGAGGATAAGGAGAGGTGAGGCAATTGAAGAGGCGAAGCTTAGCGAGTTCCAGCAAGAGCTGGTGCAGCTGGCGGCGGTGCTGAAGGGGGATCATATCCTCACAAGTTACCCGGAAAGGATAGGGAAGGACATGAGCGTGAAGGAAGGTAAAGAGTACATGGAAGATGCAGTCAAACGGTTCTTCGAGGCTGGCCATTATGCTAAGAAAATGGGAGTCGACGGCGAACAGATTGTTCAAATGAAGCCTTCCCTAACTACTCGTTCATCAAAACCTTCATCTCAACATCCATAA

>GhNPC2b

ATGTTCAAACCTGCAAGCACCGCCATCTTCTTCTTCTTCTTCAGCTGCCATGGCGGTCCAATCAAAACTATTGTGGTCCTAGTGATGGAGAATCGCTCGTTCGATCACATGCTAGGATGGATGAAGAAAATCAACCCTGAAATCAACGGTGTTGATGGAACTGAATGGAACCCTTTGTCTACCACTGATCCTAACTCCAAAAAGTTGTTCTTCCAGAACCAAGCTCAGTTCGTGGATCCAGATCCTGGTCACTCTTTCCAAGCCATAAGGGAGCAGATTTTCGGCTCTAATGACACCTCCACCAATCCTCCTCCCATGAATGGCTTCGCCCAACAAGCTTACTCCATGGACCCATCTACCACTATGTCTCAGAACGTCATGAATGGATTTGACCCTGAAATGGTCCCCGTTTACAAGTCTCTTGTCTCAGAATTTGCTGTCTTTGATCGGTGGTTCGCCTCCGTACCGTCATCCACACAACCGAATCGTCTGTACGTCCACTCGGCGACGTCGGCGGGGGCGACGAGCAACATTCCGGCACTCCTGGTAAAGGGTTATCCCCAAAGGACGATCTTCGAGAACCTGGACGCCGCCGGAATATCCTGGGGAATATACTACCAGAACATCCCAGCTACATTGTTCTACAAAAACCTCAGGAAACTCAAATACTTATTTAGATTCCGTCCGTACGGTGTGACATTCAAAAAACACGCGCAAGAAGGGAAGCTGCCGGGCTACGTTGTGGTGGAGCAGCGGTACATGGACACTAAGCTGGAGCCCGCCAACGATGACCATCCGTCGCACGATGTGTATCAAGGGCAGATGTTTGTGAAGGAGGTGTACGAGACATTGAGGGCTAGCCCGCAGTGGAACCAAACTTTGTTGATCATCACATATGATGAACATGGTGGGTTTTATGATCATGTGGCTACGCCCGTCACTGGAGTGCCCAGCCCTGATGGCATTGTGGGTCCCGAACCATTTTTCTTTCATTTCGACAGATTGGGGGTTAGGGTTCCGACCATCATGGTCTCTTCTTGGATTGATAAGGGCACTGTTGTTCATGGGGCAAATGGAAGGCCATTTCCCACATCGGAATTCGAGCATTCCTCCATTCCGGCAACAGTAAAGTTGTTGTTCAACCTTACCTCTCCATTCCTCACCAAGAGGGACGAATGGGCTGCCACTTTTGAGTCCATTCTCCGAACTCGGTCTGACCCCAGAACTGATTGTCCAGAGACACTGCCAACGCCGGCGAGGATAAGGAGAGGTGAGGCAAATGAAGAGGCGAAGCCGAGCGAGTTCCAGCAAGAGCTGGTGCAGCTGGCGGCGGTGCTGAAGGGGGATTATATCCTCACAAGTTACCCGGAAAGGATTGGGAAGGAAATGAGCGTCAAGGAAGGTAAAGAGTACATGGAAGATGCAGTCAAACGATTCTTCGAGGCTGGCCATTTTGCTAAGAAAATGGGAGTCGACGGCGAACACATTGTTCAAATGAAGCCTTCCCTAACTACTCGTTCATCAAAACCTTCATCTCAACATCCATAA

>GhNPC3a

ATGGCAGTTGAAACAAGCTCTGCAACTCCATCTCCAGTCAAAACAGTGGTTGTTTTGGTTCAAGAGAACCGTTCCTTTGACCACATGTTAGGCTGGTTCAAAACTATAAACCCAGAAATCGACGGTGTCACAGGCTCCGAATCCAACCCCATTTCCACCTCCGACCCCAACTCCACCCAAATCACCTTCAAGGACACCGCCGGCTACGTTGATCCCGACCCCGACCACTCTTTCCAAGCCATATACGAACAGGTATCCGGCAAAACGTGGGATACCAGCAACCCGGATCCGAACCCGGGGATAAAAATGAACGGTTTTGTACAAAATGCTGAACGTACAACTCCGGGGCTGTCGGAGACCGTAATGAATGGGTTTAAACCGGAGGCTGTGCCGGTGTTTAAGCAGCTAGTGACGGAGTTCGCAGTGTGTGATCGGTGGTTTGCGTCGTTGCCGGCGTCGACGCAGCCTAACAGGCTTTACGTACACTCAGCGACATCGCATGGTGCCATGAGCAACAACACACAACAGCTTATCGAAGGATTTCCTCAAAAAACTATATTTGAATCATTGGAAGAGAATGGATATAGCTTTGGGATTTATTATCAATCTTTTCCATCTACGCTTTTTTACAGGAAGCTTAGGCACTTGAAATATGTGGACAATTTCCATCAATACGATCTAAGCTTCAAGCGTCACTGTAAGGATGGGAAGCTACCAAATTATGTGGTGATTGAGCCCAGATATTTTGACATTTTAACAGCTGCTGCAAACGACGACCACCCTTCCCATGACGTCTCAGAGGGCCAGAAGCTTGTGAAGGAAATCTATGAAGCGCTCAGATCAAGTCCTCAATGGAATGAAATCTTGTTCCTGGTCATATATGATGAACATGGTGGTTTCTATGACCATGTTCCGACACCAACCGGAGTCCCTAGCCCCGATGATATTGTCGGTCCTGAGCCTTATAACTTCAAGTTTGATCGTCTTGGTTGCAGGGTTCCTGCCATTATGGTTTCCCCTTGGATTGAGCCTGGAACAGTGTTGCATAGGCCATCAGGGCCAGATCCTACATCAGAGTTCGAGCATTCCTCCATTGCAGCAACACTTAAGAAGATTTTCAATCTCAAAGAATTTCTAACAAAGCGTGATGCGTGGGCTGGTTCCTTTGATATTGTTGTCAATCGAAGCACCCCAAGAACAGACTGTCCAGAAAAACTGGCAGAGCCAGTGAAAATGAGAGACAGTGATGCAAAAGAAACAGCAAAACTAAGCGATTTTCAAGAAGAGCTAGTGCAAGCAGCAGCAGCATTGAAAGGAGATCCATTCAATCTTGTCGAAAACATGACAGTTTCATCTGGTCTCAAGTACGTTGAAGATGCCTTCAAAAAATTCTATGATGACGGCCAGAAAGCTAAGGAAATCAATGAAGTTGAAGATACTGTTTCAGCTGATGCATCAACTAGGAGGACAACAGCTTCCAAAACTTTCATGCAGAAAGTTTTCTCCTGTTTGGTTTGTGATCGTTGA

>GhNPC3b

ATGGTTTCATTAATTAATATCAGGAAGCTTAGGCACTTGAAATATGTGGACAATTTCCATCAATACGATCTAAGCTTCAAGCGTCACTGTAAGGATGGGAAGCTACCAAATTATGTGGTGATTGAGCCCAGATATTTTGACATTTTAACAGCTGCTGCAAACGAGGACCACCCTTCCCATGACGTCTCAGAGGGCCAGAAGCTTGTGAAGGAAATCTATGAAGCGCTCAGATCAAGTCCTCAATGGAATGAAATCTTGTTCCTGGTCATATATGATGAACATGGTGGTTTCTATGACCATGTTCCGACACCAACCGGAGTCCCTAGCCCCGATGATATTGTCGGTCCTGAGCCTTATAACTTCAAGTTTGATCGTCTTGGTTGCAGGGTTCCTGCCATTATGGTTTCCCCTTGGATTGAGCCTGGAACAGTGTTGCATAGGCCATCAGGGCCAGATCCTACATCAGAGTTCGAGCATTCCTCCATTGCAGCAACACTTAAGAAGATTTTCAATCTCAAAGAATTTCTAACAAAGCGTGATGCGTGGGCTGGTTCCTTTGATATTGTTGTCAATCGAAGCACCCCAAGAACGGACTGTCCAGAAAAACTGGCAGATCCAGTGAAAATGAGAGACACTGATGCAAAAGAAACAGCAAAACTAAGGGATTTTCAAGAAGAGCTAGTGCAAGCAGCAGCAGCATTGAAAGGAGATCCATTCAATCTTGTCGAAAACATGACGGTCTCATCTGGTCTCAAGTACGTTGAAGATGCCTTCAAAAAATTCTATGATGACGGCCAGAAAGCTAAGGAAATTAATGAAGTTGAAGATACTGTTTCAGCTGATGCATCAACTAGGAGGACAACAGCTTCCAAAACTTTCATGCAAAAAGTTTTCTCCTGTTTGGTTTGTGATCGTTGA

>GhNPC4

ATGTATGTATATATATCTCAACCCATTCCAAACCAGCAAAGAACATACAAAAACAGAACAATGGTTTCCCAAGGCAGCAATAGTGCGTCTTCGTATCCTATCAAAACCATAGTGATATTGGTCCAAGAAAACCGCTCATTCGATCACATGCTAGGCTGGTTCAAGTCCCTAAACCCTGAAATCGATGGCGTTACCGGATCCGAATCTAACCCCATTTCCACATCCGACCCGAACTCACCCATGGTCTTCTTCAAAGACAATTCCGAGTACGTAGACCCTGACCCTGCTCACTCCATCCAAGCCATTTACGAGCAAGTATTCGGCCACCCTTGGAGCTCTGACCTCCCTAACCCACCCCATGAACCCACAATGAATGGGTTCGCTCAAAACGCTGAGAGGACCGAAAAGGGAATGGCAGAGGCAGTGATGAAAGGGTTTAAACCTGATGCGGTGCCTGTTTACAAAGAGCTGGCGTCCAAGTTCGGAATATGTGACCGGTGGTTCGCGTCGGTGCCGGCTTCAACGCAACCGAACCGGATGTTTGTGCATTCAGCTACATCGTATGGACAGGAGAGCAATGACGCAATAAAGCTGATCAAAGGGTTTCCTCAAAAGACAATATTCGAGTCATTGGATGAAAGTGGTTTTAGTTTTGGGATATATTATCAATACCCTCCCTCCACCTTGTTCTTCAGGAACCTTAGACAAATGAAGTACTTAAAAAACTTTCATCAATTTGATCTGCACTTCAAGAAACATTGTGAAGAAGGGAAGCTTCCAAACTACGTGGTCGTCGAACAACGATACTTTGACCTCTTGTCGGTGCCTGCGAACGACGATCATCCGTCCCATGATGTCTCAGAAGGACAAAAATTTGTTAAGCAAGTATACGAGGCACTACGAAGTAGCCCCCAGTGGAAAGAAATGTTGTTGGTAATCACATATGATGAACATGGTGGATTTTATGACCATGTTCCAACACCTACAAATGGGGTCCCTAGCCCTGATGATATTGTTGGTCCTGAACCTTATCATTTCAAGTTTGATAGGCTTGGTGTTAGGGTTCCTACATTTTTTGTTTCTCCATGGATTGAACCTGGAACTGGTAAGTCTAGGGAGTCTCCATTACTGTTGTCGTTTAACTTGGGTTATTTAGATCATGTGTGTATGGTAATGGAATTTGCAGTGATACATAGGCCTTTAGGCCCATATCCTACTTCACAATTTGAGCATTCATCAATTCCTGCAACTGTCAAAAAGATTTTCAACCTGAAAGAGTTCCTAACAAAGCGTGATGCTTGGGCTGCTACTTTTGAAGGTGTTATAAACAGGAAGAACCCAAGAGTAGATTGTCCTGTTACATTACCCGAACCGGTGAAGATGAGACCTACTGAAGCAAAAGAGACGGCGAAATTAAGTGATTTCCAAAAAGAATTAGTACAAATGGCAGCAGTGCTGAATGGAGACCATAAAAGTGACATGTATCCACACAAACTTGTGGAGAAGATGACAGTTGCAGAGGCTGCCAAGTATGTGAATGGTGCTTTCAATAAGTTCTGTGATGAATGCCAAAGGGGAGGGATCCATGAATCTGAGATTGTTGAATTAGGAAAACAAGTTGAAAGGCCAAAAGGCAGATCTTTTATTTACAAGTTTTTTAAATGTCTTGTCTGCCATGATTGA

>GhNPC6a

ATGGGGGAATCCAAAGCCAGTCCACCACCTTCATTTTCCTTCATTTTCTCACTGTTTCTCACTGTTGCATGCCTCTTTACACCATTGGGAGCTCAACAGCAGTCACCCATCAAGACCATAGTGGTGTTGGTGATGGAAAACAGATCCTTTGACCACATGCTTGGGTGGATGAAGCAACACGTCAACCCTTCAATCAATGGAGTAACTGGAGATGAATGCAACCCGGTTTCAACAAAAAACCCGAACCCGGAATCCATATGCTTCACCGACGATGCTGAGTTTGTGGATCCGGATCCAGGTCACTCTTTTGAAGCTGTTGAACAACAGGTGTTTGGTTCTTCCACCATTCCTTCCATGTCTGGTTTTGTGGAACAAGCATTGTCAATGTCCAAGAACCTGTCTGAGACTGTAATGAAAGGGTTTAGACCCGAGTCAGTCCCGGTTTACGCTGCTCTCGTGAAGGAATTTGCAGTTTTCGACCGGTGGTTCTCTTCAATCCCCGGTCCAACCCAACCCAATAGACTCTTCGTTTATTCAGCCACTTCCCATGGTTCAACCAGCCATGTTAAGAAACAGTTAGCCCATGGCTACCCACAAAAAACAATCTTTGACTCTCTCCATGAGAATGATAAGGATTTTGGGGTTTATTTCCAAAACATACCCACAACTTTGTTCTATAGAAACCTTAGGAAATTGAAGTATGTTTTCAAGTTCCATCAGTTCGATTTGAAGTTCAAGAAAGATGCTAGGAAAGGTAAGCTTCCTAGCTTGACGGTGATCGAACCGAGGTATTTCGATCTTAAAGGATTGCCTGCAAATGATGATCATCCATCCCATGATGTTGCTAATGGCCAAAAGCTTGTTAAAGAGGTGTACGAGATATTGAGGGCAAGCCCTCAATGGAACCAGACACTTTTGGTGATTACTTACGATGAACACGGTGGATTCTATGACCATGTCCACACCCCATACATCAATGTCCCAAGCCCAGATGGGAACACCGGTCCGGCTCCTTCTTTCTTCAAGTTCGATAGGCTCGGTGTTCGAGTGCCGACGATCATGGTCTCTCCTTGGATCAAGAAAGGCACTGTGATTAGTGGTCCGAAGGGTCCTTTCCCGAACTCTGAATTCGAGCACTCATCGATCCCTGCAACGATAAAGAAAATGTTCAATCTATCTTCCAACTTCTTGACTCATAGAGATGCTTGGGCCGGCACTTTCGAACGCGTTGTCGGAGAGCTATCCTCTCCTAGAACTGATTGCCCAGAGAAGTTACCGGAGGCAGCACCACTGAGGACAACAGCAGCAAACGAAGACGCTGGTCTCTCTGAATTTCAGAGCGAGGTAGTTCAACTAGCCAGTGTGCTTAATGGTGACCACTTCTTGAGCGGCTTTGCCGAGGAGATGCACACAAAGATGAGCGTAAAAGGAGCTCATGAGTACGTGAAAGGCGCAGTTTCAAGGTTTATAAGAGCAAGCAAAGAGGCCATAAAGTTGGGAGCCGATGAATCTGCCATTGTTGATATGAGATCATCGCTTACAACTCGATCTTCTTCGATCCACAACTAG

>GhNPC6b

ATGGGGGAATCCAAAACCAGTCCACCACCTTCATTTTCCTTCATTTTCTCACTGTTTCTCACTGTTGCATGCCTCTTTACACCATTGGGAGCTCAACAGCAGTCACCCATCAAGACCATAGTGGTGTTGGTGATGGAAAACAGATCCTTTGATCACATGCTTGGGTGGATGAAGCAACACGTCAACCCTTCAATCAATGGAGTAACTGGAGATGAATGCAACCCGGTTTCAACAAAAAACCCGAACCCGGAATCCATTTGCTTCACCGACGATGCTGAGTTTGTGGATCCGGATCCAGGTCACTCTTTCGAAGCTGTTGAACAACAGGTGTTTGGTTCTTCCACCATTCCTTCCATGTCTGGTTTTGTGGAACAAGCATTGTCAATGTCCAAGAACCTGTCTGAGACTGTAATGAAAGGGTTTAGACCCGAGTCAGTCCCGGTTTACGCTGCTCTCATGAAGGAATTTGCAGTTTTCGACCGGTGGTTCTCTTCAATCCCCGGTCCAACCCAACCCAATAGACTCTTCGTTTATTCAGCCACTTCCCATGGTTCAACCAGCCATGTTAAGAAACAGTTAGCCCATGGCTACCCACAAAAAACAATCTTTGACTCTCTCCATGAGAATGGTAAGGATTTTGGAGTTTATTTCCAAAACATACCCACAACTTTGTTCTATAGAAGCCTTAGGAAATTGAAGTATGTTTTCAAGTTCCATCAGTTCGATTTGAAGTTCAAGAAAGATGCTAGGAAAGGTAAGCTTCCTAGCTTGACGGTGATCGAACCGAGGTATTTCGATCTTAAAGGATTGCCTGCGAATGATGATCATCCGTCCCATGATGTTGCTAATGGCCAAAAGCTTGTTAAAGAGGTGTACGAGATATTGAGGGCAAGCCCTCAGTGGAACCAGACACTTTTGGTGATTACTTATGATGAACACGGTGGATTCTATGACCATGTCCACACCCCATACATCAATGTCCCAAGCCCAGATGGGAACACCGGTCCTGCTCCTTCTTTCTTCAAGTTCGATAGGCTCGGTGTTCGAGTGCCGACGATCATGGTCTCTCCTTGGATCAAGAAAGGCACTGTGATTAGTGGTCCGAAGGGTCCTTTCCCGAACTCCGAATTCGAGCACTTGTCGATCCCTGCAACGATAAAGAAAATGTTCAATCTATCGTCCAACTTCTTGACTCATAGAGATGCTTGGGCCGGCACTTTCGAACACGTTGTCGGAGAGCTATCCTCTCCTAGAACTGATTGCCCAGAGAAGTTACCGGAAGCAGCACTACTGAGGACAACAGAAGCAAACGAAGACGCTGGTCTCTCTGAATTTCAGAGCGAGGTGGTTCAACTAGCCAGTGTGCTTAATGGTGACCACTTCTTGAGCAGCTTTGCCGAGGAGATGCGCACAAAGATGAGCGTAAAAGGAGCTCATGAGTACGTGAAAGGCGCAGTTTCAAGGTTTATAAGAGCAAGCAAAGAGGCCATAAAGTTGGGAGCCAATGAATCCACCATTGTTGATATGAGATCATCGCTTACAACTCGATCTTCTTCGATCCACAACTAG

>GhNPC6c

ATGGAGGGCTCATTTTCATTCATTTTCTTGCTGTTTCTCTTACCATTTGTAGTATCCCAAGGGTCGCCCATTAAGACAATAGTGGTTTTGGTAATGGAAAACAGATCCTTTGATCACATGGTTGGTTGGATGAAACAAAGCATAAACCCAACCATCAACGGTGTAACTGGTAATGAATGTAACCCCATTTCAACCAAAACTCCAAACCCAAAATCCATTTGTTTCACTAATGATGCTCAGTTCGTAGATCCAGATCCGGGTCATTCTTTTGAAGCTGTTGAACAACAGGTATTTGGTTCAACTCTCTCCTCATTCCCTTCCATGTCTGGTTTTGTAGAACAAGCCTTCTCAATCTCCCCAAATATGTCTGAAACAGTCATGAAAGGTTTCAAACCAGAAGCTGTCCCTATTTATGCAACATTAGTTAAAGAATTTGCTGTGTTTGATCGTTGGTTTTCATCAATCCCTGGTCCAACACAACCTAATAGACTCTTTGTTTATTCAGCTACTTCCCATGGTTCAACTAGCCATGTCAAAAAACAATTAGCACAAGGGTACCCTCAAAAAACAATCTTTGATTCACTTCATGAAAATGGCAAAGATTTTGGGGTTTATTTCCAGAATATACCCACAACTTTGTTTTATAGAAACCTTAGGAAACTCAAATATGTGTTTAAGTTCCATCAATTTGATTTGAAGTTTAAAAAAGATGCCTTGAATGGCAAGTTACCTAGCTTGAGTGTGATTGAACCAAGGTATTTTGATCTTAAAGGGTTACCTGCTAATGATGATCATCCATCACATGATGTGGCTAATGGTCAAAAGCTGGTCAAAGAAGTGTATGAGACATTGAGGGCAAGCCCTCAGTGGAACGAAACATTGTTGGTGATTACTTATGATGAACATGGTGGGTTTTATGATCATGTTAAGACACCATTTGTTAATGTTCCAAACCCTGATGGGAACACTGGTCCTGCTCCTTCTTTCTTCAAGTTTGATAGACTTGGTGTTCGTGTTCCTACTATTATGGTCTCTCCTTGGATCAAGAAAGGCACTGTGATAAGTGGTCCAAAAGGACCAACACCAAACTCAGAATTTGAGCACTCATCAATCCCTGCAACAATAAAGAAAATCTTCAACCTTTCTTCCAATTTCTTAACTCACAGAGATGCTTGGGCCGGCACTTTTGAAGATGTTGTTTCCCACTTAACTTCCCCAAGAACTGATTGTCCAGAAACATTGCCAGATGTTGTACCTTTGAGGGCAACTGAAGCAAAAGAAGATGCTGCTCTGTCTGAGTTTCAAAGTGAGGTTGTTCAACTAGCTGCTGTTCTTAACGGTGACCATTTCTTGAGCAGTTTTCCCGACGAGATGAGCAAGAAAATGACGGTGAAAGAAGCTCATGAGTACACCAAAGGGGCTATTTCCCGGTTCATACGAGCAAGTAAAGAGGCCCTCAAGTTGGGAGCTGCCGAATCTGCCATTGTAGATATGCGATCATCGCTTACAACGAGATCTTCGAATCCGTAA

>GhNPC6d

ATGGAGCGCTCATTTTCCTTCATTTTCTTGCTGTTTATCTTACCATTTGTAGTATCCCAAGAGTCACCCATTAAGACAATAGTGGTTTTGGTAATGGAAAACAGATCCTTTGATCACATGGTTGGTTGGATGAAACAAGGCATAAACCCAACCATCAATGGTGTAACTGGTAATGAATGTAACCCCATTTCAACCAAAACCCCAAACCCAAAATCCATTTGTTTCACTAATGATGCTCAGTTCGTAGATCCAGATCCAGGTCATTCTTTTGAAGCTGTTGAACAACAGGTATTTGGTTCAACTCCCTCCTCATTCCCTTCCATGTCTGGTTTTGTAGAACAAGCCTTCTCAATCTCCCCAAACATGTCTGAAACAGTCATGAAAGGTTTCAGACCAGAAGCTGTCCCTATTTATGCATCATTAGTTAAAGAATTTGCTGTGTTTGATCGTTGGTTTTCATCAATCCCTGGTCCAACACAACCTAATAGACTCTTTGTTTATTCAGCTACTTCCCATGGTTCAACTAGCCATGTCAAAAAACAATTAGCGCAAGGGTACCCTCAAAAAACAATCTTTGATTCACTTCATGAAAATGGTAAAGATTTTGGGGTTTATTTCCAAAATATACCCACAACTTTGTTTTATAGAAACCTTAGGAAACTCAAATATGTGTTTAAGTTCCATCAATTTGATTTGAAGTTTAAAAAAGATGCCTTGAATGGCAAGTTACCTAGCTTGAGTGTGATTGAACCAAGGTATTTTGATCTTAAAGGGTTACCTGCTAATGATGATCATCCATCACATGATGTGGCTAATGGTCAAAAGTTGGTCAAAGAAGTGTATGAGACATTGAGGGCAAGCCCTCAATGGAACGAAACATTGTTGGTGATTACTTATGATGAACATGGTGGGTTTTATGATCATGTTAAGACACCATTTGTTAATGTTCCAAACCCTGATGGGAACACTGGTCCTGCTCCTTCTTTCTTCAAGTTTGATAGACTTGGTGTTCGTGTTCCTACTATTATGGTCTCTCCTTGGATCAAGAAAGGCACTGTGATAAGTGGTCCAAAAGGACCAACACCAAACTCAGAATTTGAGCACTCATCAATCCCTGCAACAATAAAGAAAATCTTCAACCTTTCTTCCAATTTCTTAACTCACAGAGATGCTTGGGCCGGCACTTTTGAAGATGTTGTTTCCCACTTAACTTCCCCAAGAACTGATTGTCCAGAAACATTGCCAGATGTTGTACCTTTGAGGACAACTGAAGCAAAAGAAGATGCTGCTCTGTCTGAGTTTCAAAGTGAGGTTGTTCAACTAGCTGCTGTTCTTAACGGTGACCATTTCTTGAGCAGTTTTCCCGATGAGATGAGCAAGAAAATGACGGTGAAAGAAGCTCATGAGTACACCAAAGGGGCCGTTTCCCGGTTCATACGAGCAAGTAAAGAGGCCCTCAAGTTGGGAGCTGCCGAATCTGCCATTGTAGATATGCGATCATCGCTTACAACGAGATCTTCGAATCCGTAA
