# Supplementary material for: Structural, expression and evolutionary analysis of the non-specific phospholipase C gene family in Gossypium hirsutum
Source: BMC Genomics. 2017 Dec 19;18:979. doi: 10.1186/s12864-017-4370-6 (PMC5738194; doi:10.1186/s12864-017-4370-6)
Supplement: Supplementary file 5 — The K a/Ks ratio of four G. hirsutum duplicated gene pairs (DOC 30 kb) [file 12864_2017_4370_MOESM5_ESM.doc]

**Additional File 5: Table S3** The *K_a_*/*Ks* ratio of four *G. hirsutum* duplicated gene pairs.

| Duplicated gene pairs | *K_a_* | *Ks* | *K_a_*/*Ks* |
| --- | --- | --- | --- |
| *GhNPC1a* and *GhNPC1b* | 0.005418 | 0.035457 | 0.152794 |
| *GhNPC2a* and *GhNPC2b* | 0.011891 | 0.034267 | 0.346999 |
| *GhNPC6a* and *GhNPC6b* | 0.011682 | 0.046058 | 0.253628 |
| *GhNPC6c* and *GhNPC6d* | 0.007672 | 0.038328 | 0.200170 |
