# Supplementary material for: Structural, expression and evolutionary analysis of the non-specific phospholipase C gene family in Gossypium hirsutum
Source: BMC Genomics. 2017 Dec 19;18:979. doi: 10.1186/s12864-017-4370-6 (PMC5738194; doi:10.1186/s12864-017-4370-6)

**Additional File7: Table S4** Functional annotations of GhNPCs.

| Gene name | Gene Ontology (GO) Items | | KEGG Pathway Items | | InterPro Items |
| --- | --- | --- | --- | --- | --- |
|  | molecular_function | biological_process | KEGG Orthology | Associated Pathway List |  |
| *GhNPC1a* | GO:0016788; |  | K01114 | ko00564;  ko00565;  ko01100;  ko01110; | IPR007312; |
| *GhNPC1b* | GO:0016788; |  | K01114 | ko00564;  ko00565;  ko01100;  ko01110; | IPR007312; |
| *GhNPC2a* | GO:0003824;  GO:0016788; | GO:0008152 | K01114 | ko00564;  ko00565;  ko01100;  ko01110; | IPR007312; IPR017850; |
| *GhNPC2b* | GO:0003824;  GO:0016788; | GO:0008152 | K01114 | ko00564;  ko00565;  ko01100;  ko01110; | IPR007312; IPR017850; |
| *GhNPC3a* | GO:0003824;  GO:0016788; | GO:0008152 | K01114 | ko00564;  ko00565;  ko01100;  ko01110; | IPR007312; IPR017850; |
| *GhNPC3b* | GO:0016788; |  | K01114 | ko00564;  ko00565;  ko01100;  ko01110; | IPR007312; |
| *GhNPC4* | GO:0016788; |  | K01114 | ko00564;  ko00565;  ko01100;  ko01110; | IPR007312; |
| *GhNPC6a* | GO:0003824;  GO:0016788; | GO:0008152 | K01114 | ko00564;  ko00565;  ko01100;  ko01110; | IPR007312; IPR017850; |
| *GhNPC6b* | GO:0003824;  GO:0016788; | GO:0008152 | K01114 | ko00564;  ko00565;  ko01100;  ko01110; | IPR007312; IPR017850; |
| *GhNPC6c* | GO:0003824;  GO:0016788; | GO:0008152 | K01114 | ko00564;  ko00565;  ko01100;  ko01110; | IPR007312; IPR017850; |
| *GhNPC6d* | GO:0003824;  GO:0016788; | GO:0008152 | K01114 | ko00564;  ko00565;  ko01100;  ko01110; | IPR007312; IPR017850; |

GO:0003824: catalytic activity;

GO:0016788: hydrolase activity, acting on ester bonds;

[GO:0008152](http://amigo.geneontology.org/amigo/term/GO:0008152): metabolic process;

[K01114](http://www.kegg.jp/dbget-bin/www_bget?ko:K01114): phospholipase C;

[ko00564](http://www.kegg.jp/kegg-bin/show_pathway?ko00564+K01114): Glycerophospholipid metabolism;

[ko00565](http://www.kegg.jp/kegg-bin/show_pathway?ko00565+K01114): Ether lipid metabolism;

[ko01100](http://www.kegg.jp/kegg-bin/show_pathway?ko01100+K01114): Metabolic pathways;

[ko01110](http://www.kegg.jp/kegg-bin/show_pathway?ko01110+K01114): Biosynthesis of secondary metabolites;

[IPR007312](http://www.ebi.ac.uk/interpro/entry/IPR007312): Phosphoesterase;

[IPR017850](http://www.ebi.ac.uk/interpro/entry/IPR017850): Alkaline-phosphatase-like, core domain;


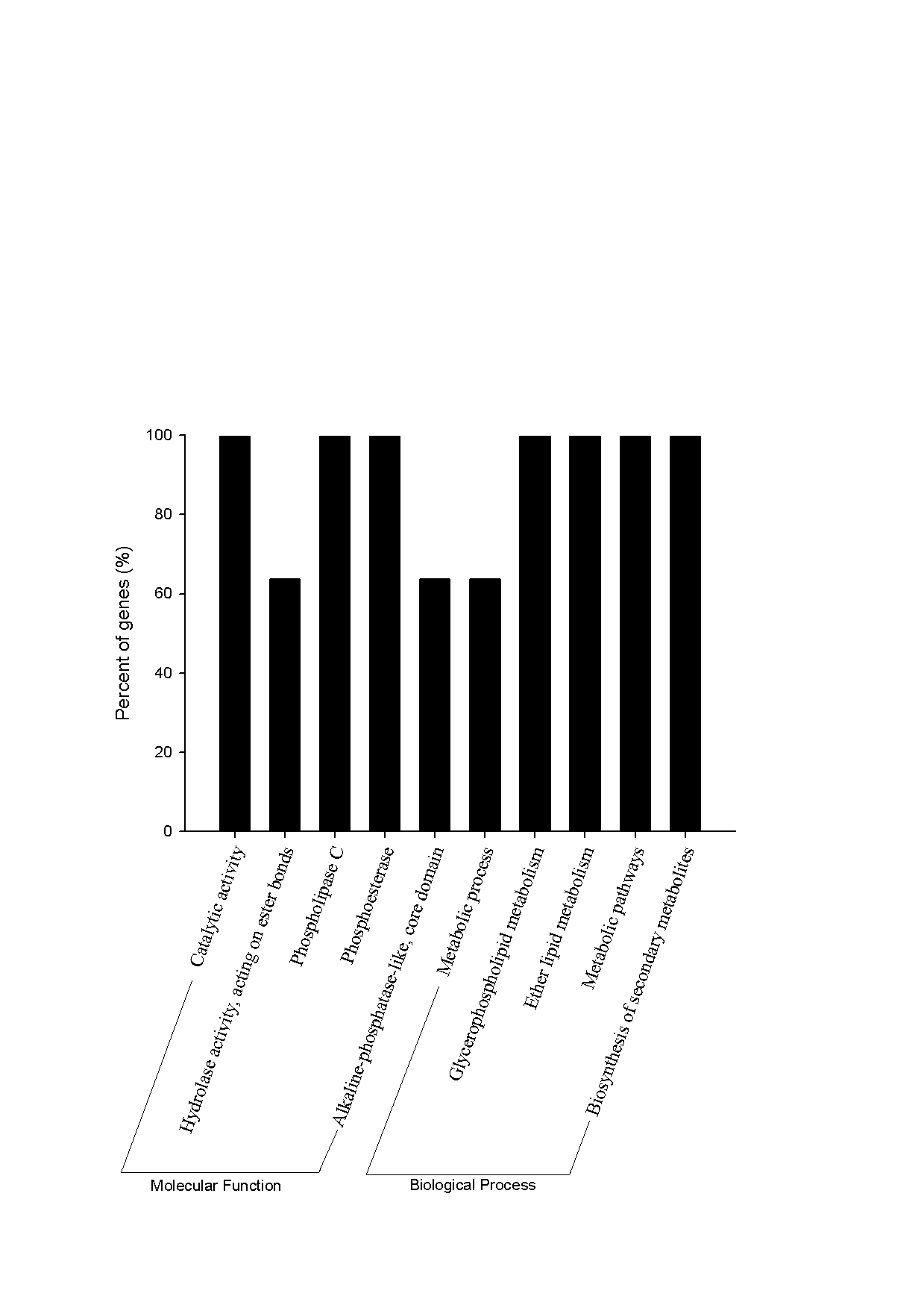

Supplement: Supplementary file 7 — Functional annotations of GhNPCs. Functional annotations of GhNPCs were predicted using the Cotton Functional Genomics Database (DOC 88 kb) [file 12864_2017_4370_MOESM7_ESM.doc]
