# Supplementary material for: Structural, expression and evolutionary analysis of the non-specific phospholipase C gene family in Gossypium hirsutum
Source: BMC Genomics. 2017 Dec 19;18:979. doi: 10.1186/s12864-017-4370-6 (PMC5738194; doi:10.1186/s12864-017-4370-6)
Supplement: Supplementary file 8 — Sequence information of twenty motifs (DOC 38 kb) [file 12864_2017_4370_MOESM8_ESM.doc]

| Name | Sequence |
| --- | --- |
| 1 | PANDDHPSHDVANGQKLVKEVYEILRASPQWNETLLVITYDEHGGFYDHV |
| 2 | YPQKTIFDSLDENGKSFGIYYQNIPTTLFYRNLRKLKYLFKFHQYDLKFK |
| 3 | TPVTGVPSPDGIVGPEPFFFKFDRLGVRVPTIMVSPWIEKG |
| 4 | AVFDRWFASIPASTQPNRLYVHSATSHGA |
| 5 | LRMSEAMSEAVLSEFQPEAVQVAAVLVGE |
| 6 | ESNPISTKDPNSKSICFTBDAZFVDPDPGHSFZAIEZQVFG |
| 7 | PKGPFPTSEFEHSSIPATVKKJFNLSS |
| 8 | NFLTKRDAWAGTFENVVKELSSPRTDCPE |
| 9 | HAREGKLPNYVVIEPRYFDLK |
| 10 | AJKLGABESAIVDMRSSLTTR |
| 11 | MNGFVZQA |
| 12 | GWLKSINPEIDGVSG |
| 13 | TSHVKKQLAHG |
| 14 | NLVENMTVSSGLKYVEDAFKKFYDDGQKA |
| 15 | LTKRDAWAGSFDIVVNRSTPRTDCPEKL |
| 16 | NRSFDH |
| 17 | IEDRGQHVEAY |
| 18 | FIFLLFJLPFVVSQESPIKTI |
| 19 | MFKPANTAIFFFF |
| 20 | YKDFKCLVC |

**Additional File 8: Table S5.** Sequence information of twenty motifs.
